# Sharp-wave ripple doublets induce complex dendritic spikes in parvalbumin interneurons in vivo

**DOI:** 10.1038/s41467-022-34520-1

**Published:** 2022-11-07

**Authors:** Linda Judák, Balázs Chiovini, Gábor Juhász, Dénes Pálfi, Zsolt Mezriczky, Zoltán Szadai, Gergely Katona, Benedek Szmola, Katalin Ócsai, Bernadett Martinecz, Anna Mihály, Ádám Dénes, Bálint Kerekes, Áron Szepesi, Gergely Szalay, István Ulbert, Zoltán Mucsi, Botond Roska, Balázs Rózsa

**Affiliations:** 1 Laboratory of 3D Functional Network and Dendritic Imaging, Institute of Experimental Medicine, Budapest, Hungary; 2 Two-Photon Measurement Technology Group, The Faculty of Information Technology, Pázmány Péter University, Budapest, Hungary; 3 Momentum Laboratory of Neuroimmunology, Institute of Experimental Medicine, Budapest, Hungary; 4 Faculty of Information Technology and Bionics, Pázmány Péter University, Budapest, Hungary; 5 Institute of Cognitive Neuroscience and Psychology, Research Centre for Natural Sciences, Budapest, Hungary; 6 BrainVisionCenter, Budapest, Hungary; 7 Institute of Molecular and Clinical Ophthalmology Basel, Basel, Switzerland; 8 Neural Circuit Laboratories, Friedrich Miescher Institute, Basel, Switzerland; 9 Department of Ophthalmology, University of Basel, Basel, Switzerland

**Keywords:** Dendritic excitability, Fluorescence imaging, Neural circuits

## Abstract

Neuronal plasticity has been shown to be causally linked to coincidence detection through dendritic spikes (dSpikes). We demonstrate the existence of SPW-R-associated, branch-specific, local dSpikes and their computational role in basal dendrites of hippocampal PV+ interneurons in awake animals. To measure the entire dendritic arbor of long thin dendrites during SPW-Rs, we used fast 3D acousto-optical imaging through an eccentric deep-brain adapter and ipsilateral local field potential recording. The regenerative calcium spike started at variable, NMDA-AMPA-dependent, hot spots and propagated in both direction with a high amplitude beyond a critical distance threshold (~150 µm) involving voltage-gated calcium channels. A supralinear dendritic summation emerged during SPW-R doublets when two successive SPW-R events coincide within a short temporal window (~150 ms), e.g., during more complex association tasks, and generated large dSpikes with an about 2.5-3-fold amplitude increase which propagated down to the soma. Our results suggest that these doublet-associated dSpikes can work as a dendritic-level temporal and spatial coincidence detector during SPW-R-related network computation in awake mice.

## Abstract (web summary)

The influence of sharp-wave ripples (SPW-Rs) on dendritic computation remains poorly understood. Here, the authors demonstrate the existence of SPW-R associated, branch-specific, dendritic spikes which serve as a temporal and spatial coincidence detectors during SPW-R-doublets in PV+ interneuron dendrites of awake mice.

## Introduction

Sharp-wave ripples (SPW-Rs) are one of the most synchronous events in the mammalian brain, playing a key role in memory encoding, consolidation, and retrieval^1–5^. Their most important feature is the temporally compressed and phase-locked firing of the same neuronal sequences that were active in theta cycles during exploratory behavior^1,3,5–7^. Recent data suggest that extended memory cues require elongated SPW-R activity coded as chains of ripples (doublets or triplets) or prolongated ripple events^8–10^. SPW-Rs are generated by an interplay between pyramidal cells and interneurons, of which parvalbumin-expressing (PV+) interneurons are key elements^6,11–17^: the SPW-R-associated cell assemblies provide synchronized inputs to the dendrites of PV+cells.

Previous in vitro models have shown that dendrites of PV+ neurons perform passive, sublinear integration during low neuronal network activity: this is associated with suppressed action potential backpropagation and lack of dendritic spikes^18–27^. Nevertheless, when neuronal network activity is artificially increased, such as in the in vitro SPW-R model, synchronous inputs “bombard” PV+ cell dendrites which, similarly to the dendrites of pyramidal cells^28–32^, “come to life”: this could generate supralinear dendritic summation, NMDA spikes, and even more broadly distributed regenerative events, dendritic Ca^2+^ spikes^23,33–39^. Accordingly, SPW-R-associated dSpikes have been discovered recently in hippocampal pyramidal cells^23,28–40^. The neuroscientific community is divided into opposing groups on the subject of interneurons: some question the existence of active dendritic computation and dSpikes in interneurons (View #1)^19,21–26,41,42^; others believe that dendrites can be active and induce regenerative events such as dSpikes and action potential (AP) backpropagation with non-decremental amplitude (View #2)^33–39,43^. According to View #1, PV+ interneurons have passive dendrites dampened by a high potassium to sodium channel ratio: these suppress dendritic regenerative activities (dSpikes and AP backpropagation), which results in sublinear dendritic integration. We could define this state as a “ground” state of the PV+ interneurons: this is the default state and plays a crucial role in neuronal computation. However, according to View #2, PV+ cells also have an “excited” state, which exists in parallel with the “ground” state: in active network states, for example during SPW-R events when PV+ interneurons are bombarded with ongoing excitatory activity, the passive, well-dampened dendrites exceed their activation threshold and can cause supralinear integration, NMDA-spikes, and propagating dendritic calcium spikes. Dendritic activity has not been mapped during in vivo SPW-Rs, where network activity is physiological: dendritic spikes have not previously been shown in PV+ cells due to technical limitations.

Our combined fast 3D imaging and local electrophysiological method has provided access to the thin distal dendritic segments of basal dendritic arbor in PV+ interneurons of awake mice, by enabling the simultaneous recording of multiple, thin dendritic segments with a total length of over 1500 µm. This has led us to discover sharp-wave-ripple-associated dendritic spikes (SPW-R-dSpikes) and an other type of neuronal computation during in vivo SPW-R doublets. In contrast to the obvious disagreement between the two views, we show here that these two integration modes, the “ground” and “excited” states, are equally important and coexist in the same interneurons. In addition, these two states are also “colocalized”: in low-activity network states, a dendrite is in the ground state of passive integration; when the number of ongoing inputs increases, the appearance of multiple hot-spots and propagating regenerative events switches the same dendrite to the excited state by.

## Results

### Recording network and dendritic activity patterns in 3D during in vivo SPW-Rs

**Fig. 1:**
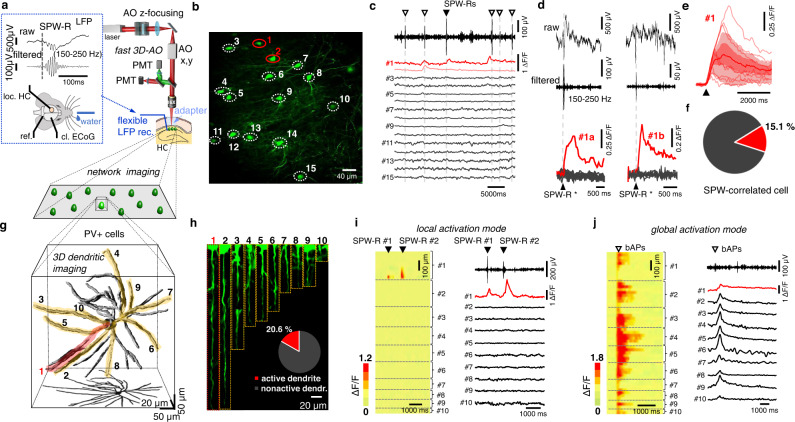
Activation of long dendritic segments revealed by fast 3D imaging in SPW-R-associated PV+ cells in behaving mice. **a** Schematic of the combined LFP recording, fast raster scanning, and 3D dendritic measurements with AO microscopy (**g**) during SPW-Rs in the periods of water licking. Inset, representative raw and filtered local hippocampal LFP signals with an SPW-R event from the stratum pyramidale from the ipsilateral hippocampus. Bottom panel indicates the position of the local hippocampal (loc. HC), reference (ref.) and contralateral cortical (cl. ECoG.). **b** Red and white dashed circles indicate PV+ neurons in the CA1 region of the hippocampus, which were active and inactive, respectively, during SPW-Rs. **c** Local LFP signal and somatic Ca^2+^ responses recorded simultaneously to **b**. Arrowheads indicate SPW-R events. Red traces indicate SPW-R-associated somatic responses. **d** Left raw, enlarged view of the SPW-R (#1a) event indicated with filled arrowhead in **c** is shown with and without filtering with corresponding somatic responses. Right raw, same as left raw, but with another event (#1b). **e** Individual SPW-R-associated Ca^2+^ transients from cell **#1** overlaid with the average (mean ± SEM). **f** Ratio of the active PV+ neurons, which showed SPW-R-correlated Ca^2+^ responses. **g**–**j** Non-uniform (global and local) dendritic activation revealed by fast 3D-AO imaging in a sample SPW-R-correlated PV+ cell under in vivo conditions. **g** Dendritic arbor of the SPW-R-correlated cell in the stratum oriens. Red and yellow ribbons indicate dendritic segments selected for fast 3D ribbon scanning. The total length of the selected and simultaneously recorded dendritic segments was 1568 µm. **h** Fluorescence was recorded simultaneously along the 10 dendritic regions shown in **g** and then projected into 2D as a function of distance along the longitudinal and transverse directions of the 3D ribbons (Supplementary Movie 1). Inset indicates ratio of recorded dendrites with SPW-R-correlated activity (*n* = 11/5, cells/mice). **i** Left, example 3D Ca^2+^ response during SPW-R events from the numbered dendrites shown in **h**. Right, average dendritic responses from the same ten dendrites. Black arrowheads indicate two SPW-R events. **j** Similar to panel **i** but during putative backpropagating action potentials. Empty arrowheads denote putative backpropagating action potentials.

Mapping the dendritic activity of PV+ interneurons is challenging, as these cells have extended dendritic arbor in 3D, with thin, tortuous, dendritic segments, which take a long time to image using classical imaging methods. Moreover, the motion inherent to awake experiments has made recording activity from these thin 3D structures impossible up to now. In addition, SPW-Rs are known to be associated with sparse activation patterns^44,45^, which further decreases the probability of detecting SPW-R-associated events in the arbor of thin dendrites. To address these technical limits, we increased the access rate to multiple long dendritic segments by using fast 3D acousto-optical imaging (Fig. 1): this enlarged the signal-to-noise ratio and measurement speed by orders of magnitude (see Equation S84 in ref. 46) compared to volume-scanning methods based on classical point-by-point scanning. It also enabled fast motion-artifact elimination at high resolution^46^. To minimize the distance between the imaged neurons and the hippocampal recording electrode, we developed a plastic, eccentric deep-brain adapter instead of using the more common metal cylinder^47,48^. We combined this with a custom-made flexible four-wire electrode for chronic hippocampal local field potential (LFP) recordings, which increased the useful area for electrode insertion, and resulted in decreased electrical noise (Supplementary Figs. 1 and 2). Moreover, we extended the fast scanning ranges of 3D AO microscopy^46,49^, which made it possible to reach the hippocampal regions under the deep-brain adapter. Then we combined simultaneous ipsilateral, local recording of the hippocampal LFP signal with fast 3D network and dendritic imaging (Fig. 1 and Supplementary Fig. 1). To reveal SPW-R-coupled dendritic activity, we labeled PV+ cells with GCaMP6f and used either the fast arbitrary frame-scanning mode of the 3D AO microscope or resonant scanning in the first step to find SPW-R-correlated somatic activity (Fig. 1a–c). Physiological and anatomical parameters (e.g., dendrite diameter; baseline fluorescence intensity; amplitude; full width at half maximum; and decay and rise times of the Ca^2+^ responses) did not change during the measurement (Supplementary Fig. 3), indicating there was no phototoxicity affecting the somatic and dendritic measurements. This may be explained by the low-power temporal oversampling (LOTOS) strategy, which is an inherent feature of fast AO scanning^46,50^. At the somatic level, only a subpopulation of PV+ cells (*n* = 86/261 cells) were active during the experiments; 15.1% of these active cells were involved in SPW-R events (Fig. 1b–f), in agreement with our previous results^51^. In the second set of experiments, we recorded *z*-stacks around the active cells. Multiple long, dendritic processes of the active cells were then fitted by ribbons and recorded simultaneously at 63.94 ± 18.43 Hz in 3D in behaving mice to investigate SPW-R-associated dendritic activities (Fig. 1g–j). 3D fluorescence data were projected into 2D following non-rigid elastic motion correction (Fig. 1g–h and Supplementary Movie 1). We imaged the full dendritic arbor in the stratum oriens of *n* = 12 CA1-PV+ interneurons from *n* = 6 mice and mapped *n* = 58 dendrites with their surrounding neuropil from the soma to the distal dendritic regions while reaching the thin (<500 nm diameter), terminal dendritic segments where SPW-R-dSpikes were found. The total length of the simultaneously recorded dendritic segments could exceed 1500 µm (Fig. 1h–j). To increase the probability of capturing SPW-R-associated dSpikes, we let water-deprived mice drink because this consummatory behavior induces SPW-Rs^5^. In summary, we combined chronic fast 3D dendritic and network imaging with local hippocampal LFP recording in water-deprived awake mice to link SPW-Rs and dendritic activity.

### Two dendritic activation modes during in vivo: global activation and SPW-R-dSpikes

**Fig. 2:**
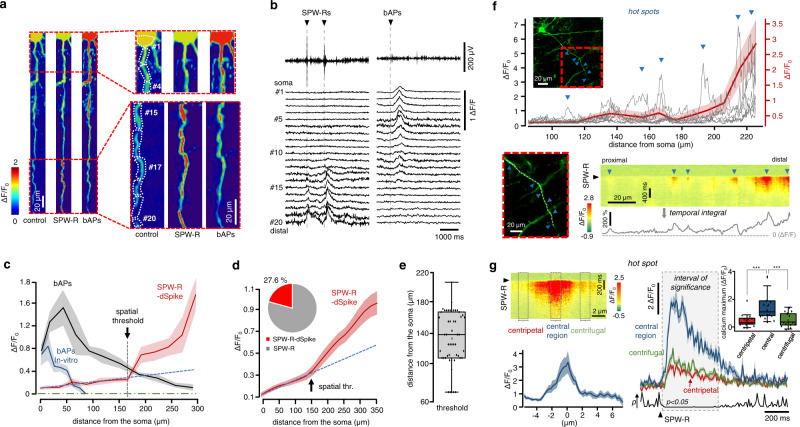
Spatial characterization of SPW-R-dSpikes in CA1 PV+ interneurons of awake mice. **a** Example dendritic 3D Ca^2+^ responses from dendrite #1 shown in Fig. 1h at rest (control), during an SPW-R event (SPW-R), and during SPW-R independent putative backpropagating APs (bAPs). **b** Individual Ca^2+^ responses spatially averaged for the numbered dendritic regions indicated in **a** show a distance-dependent decrease and increase during putative bAPs and SPW-Rs, respectively. **c** Spatial distribution of the average dendritic 3D Ca^2+^ responses from dendrite #1 during putative bAPs and SPW-R-dSpikes (mean ± SEM, *n* = 4/5 bAPs/spikes). Black arrow indicates the spatial thresholds of the spike. Blue dashed line indicates linear fit to the proximal dendritic regions. Blue transients are overlaid with averaged 3D Ca^2+^ responses recorded in vitro and were induced by 5 APs. **d** Average SPW-R-dSpike-associated dendritic 3D Ca^2+^ responses as a function of distance from the soma (*n* = 11/5 cells/mice). Blue dashed line and black arrow indicate same as on panel **c**. Inset, pie chart denotes reliability of reactivation of the dendritic spike during successive SPW-Rs (75 spikes/ 287 SPW-R events/ 11 dendrites). The average length of the recorded dendrites was 314.2 ± 43.1 µm (mean ± SD). **e** Distribution of the spatial threshold of the SPW-R-dSpikes (*n* = 11/5/51 dendrite/mice/trace). **f** Red dashed boxes show the maximal intensity z projection of the imaged dendritic regions, with two different magnifications. Top, amplitude of individual SPW-R-associated 3D Ca^2+^ responses (gray traces) recorded along the white dashed curve from a dendritic segment of a PV+ neuron. Red trace is the average response (mean ± SEM). Bottom right, an exemplified individual SPW-R-associated 3D Ca^2+^ responses as a function of time and dendritic distance with the temporal integral of the response (gray line). Blue arrowheads indicate locations with local response maxima (hot spots). **g** Left, average SPW-R-associated 3D Ca^2+^ responses from hot spot regions and their histogram (blue line, mean ± SEM; *n* = 16). Right, average SPW-R-associated Ca^2+^ transients (mean ± SEM) from the central (blue) and lateral dendritic regions (red: centripetal, green: centrifugal) of hot spots. Panel **e** and **g** inset, box-and-whisker plots show the median, 25th and 75th percentiles, range of nonoutliers and outliers. Time delay for centrifugal vs. centripetal: *p* = 0.64; for centrifugal vs. central: *p* < 0.0001, paired *t*-test).

3D ribbon scanning determined two major types of dendritic activation in PV+ cells. The first activation mode was associated with SPW-R-related events and was typically restricted to one dendritic segment with a high response amplitude in the distal dendritic region, which decreased as a function of distance toward the soma (Fig. 1i). The second dendritic activation mode occurred between SPW-R events and was represented by a global activation in all dendritic segments, with a high amplitude in the somatic and proximal dendritic domains (global activation mode, Fig. 1j). Therefore, the global events were independent of SPW-R events. The existence of a global activation mode also confirmed that all the recorded dendritic segments were capable of activity and that the local activation mode, restricted to individual dendrites, was not a result of photodamage. According to their spatial distribution profiles and temporal kinetics, we identified the local activation mode as SPW-R-associated dendritic Ca^2+^ spikes (SPW-R-dSpike) and the global activation mode as putative backpropagating action potentials (putative bAPs) independent of SPW-Rs (Figs. 1i–j and 2a–d; Supplementary Fig. 4 and Supplementary Movie 2, see Methods). Measuring 58 dendrites of 12 PV+ neurons (*n* = 6 mice, Fig. 1h), we demonstrated that 20.6% of the dendrites were active during individual SPW-R activity. In total, *n* = 85 dSpikes were detected and recorded in 3D during *n* = 307 SPW-R events, corresponding to a 27.6% “total activation ratio” (see inset in Fig. 2d and Methods). The “average activation ratio” of dSpikes (dSpikes/SPW-R event) for individual neurons was 29.9 ± 4.18% (mean ± SEM, *n* = 11 cells, *n* = 6 mice). A recent paper^51^ showed a similar (~25%) activation ratio of PV+ somata during SPW-Rs.

In our measurements, we cannot rule out the possibility that bAPs could also occur during in vivo SPW-R events and SPW-R-dSpikes. Supporting this, the amplitude of the average dendritic Ca^2+^ response during SPW-R-dSpikes was 0.17% (Δ*F*/*F*) close to the soma (at < 10 µm from the soma) (Supplementary Fig. 4b), which corresponds to about 7 APs according to the in vitro input–output calibration curve of PV+ cell dendrites (Supplementary Fig. 5b, c). Moreover, the average amplitude of the in vivo SPW-R-dSpikes close to the soma (red curve in Supplementary Fig. 4b, 0 µm = soma) was higher than the amplitude of the somatically suprathreshold SPW-R-dSpikes recorded in vitro (blue curve in Supplementary Fig. 4b), indicating that APs could be generated during in vivo SPW-R-dSpikes. In addition, somatic APs were validated by patch-clamp recordings in these in vitro measurements, further corroborating that the approximately twofold higher in vivo Ca^2+^ responses are associated with APs.

In the rest of the paper, we focus on the validation of SPW-R-dSpikes by showing the regenerative nature of these in vivo Ca^2+^ signals in multiple steps. The first proof of the regenerative nature of SPW-R-dSpikes was the existence of a spatial activation threshold. The relatively small distance-dependent rise in amplitude of the in vivo SPW-R-dSpikes was superimposed with a sharp and strong supralinear increase at a critical distance, characterized by an inflection point (defined as the spatial threshold of the spike: 133.24 ± 4.98 µm (*n* = 11/5 cells/mice; Fig. 2c–e, Supplementary Fig. 4a, b, e), which cannot be explained by either neuropil contamination (Supplementary Fig. 6) or the dendritic geometry, as the correlation between branching points and the location of the spatial thresholds was low (Supplementary Fig. 7a–d, Pearson’s *r* value = −0.24). The existence of the spatial threshold confirmed that SPW-R-dSpikes are regenerative events.

### Propagation of dendritic Ca^2+^ spikes

To further validate the regenerative nature of SPW-R-dSpikes we recorded their initialization and propagation by increasing the speed of 3D imaging to 308 ± 24 Hz (mean ± SD) and by using temporal super-resolution microscopy as previously described^49^ (Fig. 2f, g). Similarly to the in vitro case^36^, the SPW-R-dSpike originated from multiple dendritic input zones (hot spots): however, in contrast to the in vitro results, the location and amplitude of these hot spots varied between successive in vivo SPW-R events (Fig. 2f). Despite this increased variability, the spatial and temporal profiles of individual hot spots were similar in vivo (Fig. 2g, full width at half maximum: 5.41 ± 0.16 µm) and in vitro^18,42,52,53^. SPW-R-dSpikes were initiated 13.83 ± 2.46 ms (*n* = 16 hot spots, p < 0.05) earlier in centers of hot spots from which spikes propagated centripetally and centrifugally faster than 6.60 ± 1.41 µm/ms (*n* = 16, Fig. 2g). The hot spot-associated initiation and then the fast propagation also corroborated the regenerative nature of SPW-R-dSpikes.

### Mechanism of in vivo SPW-R-dSpikes

The following in vitro procedure was used to test the underlying mechanism of SPW-R-dSpikes: first, we reproduced individual glutamatergic synaptic inputs by using DNI-glutamate uncaging^36^; second, we generated spatially and temporally clustered input patterns using an input number above the dSpike threshold to mimic SPW-R-dSpikes (Supplementary Fig. 8a); third, we added different ion-channel blockers. A cocktail of voltage-gated calcium-channel (VGCC) blockers eliminated the laterally propagating spikes but was not able to eliminate the spike in the hot spot regions, these depended on α-amino-3-hydroxy-5-methyl-4-isoxazolepropionic acid (AMPA) and N-methyl-d-aspartate (NMDA) receptors in basal dendrites (Supplementary Fig. 8). Therefore, in accordance with our previous work^36^, our data indicated that the dendritic spikes can be initiated in an AMPA- and NMDA-receptor-dependent manner from hot spot regions of basal dendrites, and can then propagate in both directions utilizing, primarily, VGCC channels. In the proximal dendritic region, below the activation spatial threshold, the amplitude of the in vitro and in vivo SPW-R-dSpikes were close to each other; however, they deviated in distal dendritic regions where SPW-R-dSpikes had a much higher amplitude in vivo (Supplementary Fig. 4a, b). This is consistent with the enhanced hot spot activity in distal regions, which reflects an increased input density in vivo (Fig. 2f). Previous studies have indicated that basal dendritic segments remain silent during in vitro SPW-R events, but apical dendritic segments show VGCC-dependent Ca^2+^ responses and dSpikes^36^. In contrast, here we demonstrated that basal dendrites in the stratum oriens are also activated in vivo. We, therefore extended our previous in vitro pharmacological measurements to the entire dendritic arbor by investigating the mechanism of SPW-R-dSpikes in the basal dendrites. In conclusion, our in vitro and in vivo data confirmed that SPW-R-dSpikes are generated dominantly in distal dendritic regions by primarily utilizing regenerative NMDA and AMPA receptors, and VGCC channels.

### Multiple groups of SPW-R-associated responses in vivo

**Fig. 3:**
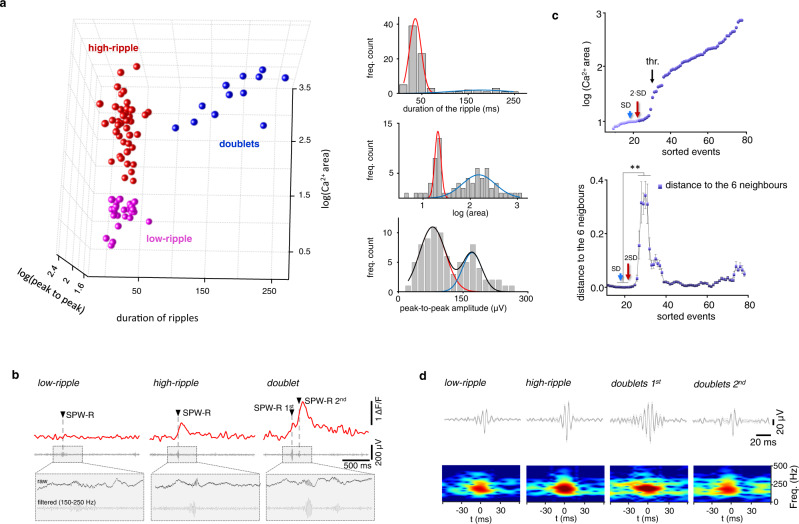
Separation of the three different types of SPW-R-associated activity. **a** Left, SPW-R-associated events plotted as a function of duration and peak-to-peak amplitudes of ripples and Ca^2+^ areas of SPW-R events (low-ripple: magenta, high-ripple: red, doublets: blue). Both the Ca^*2+*^ area and the peak-to-peak amplitude underwent logarithmic transformation. Right, spectral quantification of the three parameters shown in the left panel. Top right, frequency count of the ripple event durations separated doublets from the high-ripple + low-ripple groups. Frequency counts were fitted with two Gaussian distributions corresponding to the doublets (blue) and the high-ripple + low-ripples (red) groups. Note the gap between singlets and doublets. Middle right, same as top right panel but for Ca^2+^ areas following logarithmic transformation. The Peak Analyzer module (Origin Pro, OriginLab) identified two Gaussian distributions (*χ*^2 ^= 1.28, adj. *R*-Square = 8.30 ∙ 10^−1^, SS = 24.35) corresponding to the low-ripple (red) and the high-ripple + doublet (blue) groups. Bottom right, frequency count of peak-to-peak amplitude. The Peak Analyzer module identified two Gaussian distributions (*χ*^2^ = 1.49, adj. *R*-Square = 8.64 ∙ 10^−1^, SS = 16.38): the low-ripples (red) and high-ripples + doublets (blue). The black trace shows the sum of the two traces. See details in Methods. **b** Representative individual Ca^2+^ and associated LFP signals from the low-ripple, high-ripple, and SPW-R-doublet groups. Arrowheads indicate SPW-R events. **c** Gap statistical method. Top, areas of SPW-R-associated Ca^2+^ responses were sorted following logarithmic transformation. Data below the amplitude jump (black arrow, *thr*.) belonged to the low-ripple group in panel **a**, while data above the threshold represented high-ripple and doublet-associated responses. Bottom, average Euclidean distance (mean ± SD) from the six closest neighbors calculated from data in the top panel. Asterisks indicate statistical significance at the center four points (two-sided two-sample *t*-test, *p* = 0.00134, 2.38 ∙ 10^−7^, 3.08 ∙ 10^−6^, 0.00011). Arrows indicate noise of dendritic measurements (blue arrows: 1 SD, red arrows: 2 ∙ SD). See details in Methods. **d** Top, example filtered average LFP signals from the three groups (*n* = 15/groups randomly selected events). The first and second events of the SPW-R doublets are shown separately. Bottom, corresponding frequency spectrograms. Note the similarity between the high-ripple group and the first event of the SPW-R doublets, while the low-ripple group was similar to the second event of the SPW-R doublets.

In contrast to in vitro data^36^ (Supplementary Fig. 9), the increased temporal dynamic of the spatial distribution of the emerging hot spots (Fig. 2f) under in vivo conditions indicated that the ongoing synaptic inputs and, therefore, the underlying network activity also had an enhanced dynamic. Indeed, amplitude, length, and power of the LFP showed a higher event-to-event variability than under in vitro conditions, where SPW-R events appeared in rather stereotypic patterns^36^. To better understand the mechanisms underlying SPW-R-dSpikes, we investigated how the event-to-event variability shown at the network level by the LFP signal is reflected in dendritic responses and dSpikes. To address this question, we separated SPW-R events and the corresponding 3D dendritic responses into multiple subpopulations with standard SPW-R parameters: the peak-to-peak amplitude and the duration of the ripples and the area of Ca^2+^ responses. Each of the three spectral histograms of the parameters showed two separate peaks (Fig. 3a), which therefore separated SPW-R events and the corresponding 3D dendritic responses into three groups of responses in 3D space: the low-ripple group; the high-ripple group; and the group of SPW-R doublets (Fig. 3a, b). Separation of doublets from singlets has already been shown in previous in vivo studies^8–10^ but separation of the previously unknown low-ripple and high-ripple groups was further reinforced by using gap statistical analysis: the distance metric showed a significant increase at the gap between the two groups (Fig. 3c). The three groups of SPW-R-associated responses were also validated by *K*-means cluster analysis (Methods) and by t-distributed stochastic neighbor embedding cluster analysis on an extended database (Supplementary Fig. 10a). In addition, a distance function generated from six SPW-R-associated parameters was also able to separate the same three groups of responses when it was used in sequence with cluster analysis Levenberg-Marquardt algorithm (LMA), *p* = 4.07*10^−5^ (Supplementary Fig. 10 and Supplementary Methods).

**Fig. 4:**
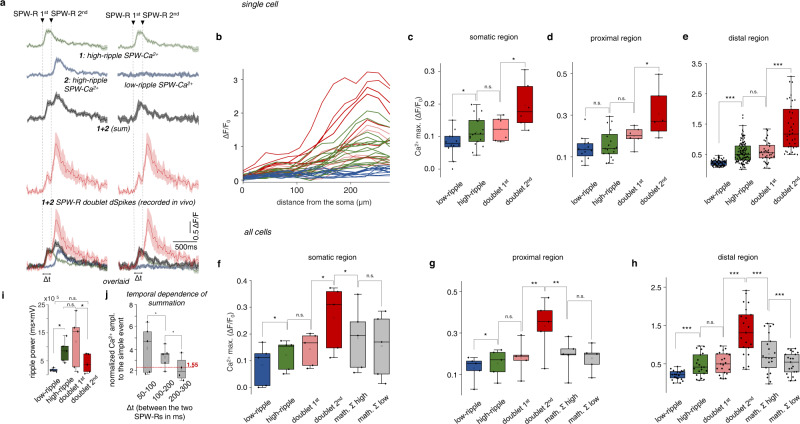
Supralinear dendritic Ca^2+^ signal integration during in vivo SPW-R-doublets. **a** Non-linear summation of the 3D Ca^2+^ responses during SPW-R doublets were calculated using two different approaches in a distal dendritic segment of a PV+ neuron (above the spatial threshold in the range of 134-300 µm from the soma). Left column, green and blue transients are average dendritic Ca^2+^ responses from the high-ripple group. The blue transient was shifted with the average time interval of doublets. Red and black transients are the average response recorded during SPW-R doublets and the mathematical sum, respectively. Bottom, the overlaid average transients indicate strong supralinear integration. Right column, the same calculation, but the second transient (blue) was replaced by the average response from the low-ripple group because the second event of the doublets statistically belongs to the low-ripple group (Figs. 3d and 4i). **b**–**e** Somato-dendritic distribution (0–285 µm) of SPW-R-associated dendritic activity along the somato-dendritic axis during low-ripple (blue), high-ripple (green), and the first (pink) and second (red) peaks of doublets in an example PV+ interneuron (*n* = 18/12/6 high ripples/low ripples/doublets). **b** Distribution of individual SPW-R-coupled Ca^2+^ transients. Data were recorded at submicron resolution using 3D ribbon scanning to precisely follow thin dendritic segments and then binned into 25 µm intervals. In contrast to low-ripple-associated responses, high-ripple- and doublet-associated Ca^2+^ transients increased as a function of distance from the soma. **c** Box-and-whisker plots of the amplitude of the transients showing the coactivation at the somatic (0 µm) compartment. **d** The same but for proximal dendritic segments (<20 µm), **e** The sam**e** but for distal (>150 µm) dendritic segments (Student’s unpaired *t*-test; *p < 0.05, **p < 0.01, ***p < 0.001). **f**–**h**, Box-and-whisker plots of the amplitude of SPW-R-coupled Ca^2+^ transients from all PV+ cells in the somatic, proximal and distal dendritic regions (*n* = 5 PV+ cells, *n* = 33 somato-dendritic segments, *n* = 51 SPW-R-dSpikes, *n* = 409 somato-dendritic segment-related SPW-R-Ca^2+^ events). The mathematical sum of two high-ripple-associated responses (*math. Σ high*), and one low- plus one high-ripple-associated response (*math. Σ low*) were calculated with 100 ms delay between the events of doublets. **i** Box-and-whisker plots of the power of the simultaneously recorded SPW-R events (*n* = 5 cells/mice). **j** Temporal dependence of the amplitude of summation during doublets.

In the low-ripple group, the amplitude and power of the ripple oscillation and the dendritic Ca^2+^ responses were significantly lower than in the high-ripple group (both SPW-R peak and Ca^2+^: *p* < 0.001; Figs. 3b, d and 4a–i; Supplementary Fig. 12 a–d, g). In the group of SPW-R doublets (20.8% of the total events, *n* = 77/51/16, SPW-R correlated event/dspike/doublet), two SPW-R events followed each other with 145.76 ± 18.77 ms temporal delay and dendritic responses were 8.82-fold and 2.92-fold higher than in the low- and high-ripple groups, respectively (Fig. 4a–h).

We averaged the population data across individual neurons and mice (Fig. 3a), revealing a high level of stability and uniformity in the differences between the low-ripple, high-ripple and doublet groups (Supplementary Fig. 10b–d).

### Dendritic coincidence detection during SPW-Rs

Although SPW-R doublets have been identified in previous studies^8–10^, and represent about one fifth of the SPW-R events in this work, little is known about them: for example, doublet-associated Ca^2+^ responses have not yet been demonstrated. The very high amplitude of the SPW-R-dSpikes during doublets in the distal dendritic region (Fig. 3 and Supplementary Fig. 12) may indicate supralinear summation and, hence, an other form of coincidence detection in CA1 hippocampal PV+ interneurons: this is found when two SPW-R events occur within a short temporal window. Indeed, the high responses during SPW-R doublets (Fig. 4a, left column) could not be explained by the linear summation of two SPW-R-dSpikes because the sum of two average dendritic Ca^2+^responses from the high-ripple group (following a time shift with the average temporal delay between the two events of doublets) was only 51.58 ± 5.44% of the average dendritic response measured during SPW-R doublets in the distal dendritic region of PV+ neurons (Fig. 4a–h). Although the second ripple event could have a higher amplitude and power than the first event (Supplementary Fig. 13a–c), on average the first event had significantly higher ripple power and the second event had higher dendritic responses (Ca^2+^: *p* < 0.001, SPW-R power: *p* = 0.05; Fig. 4b–i). Moreover, according to the LFP power, the first event belonged statistically to the high-ripple group (SPW-R power: *p* = 0.32; Ca^2+^: *p* > 0.22; Fig. 4b–h and Supplementary Fig. 14), and the second event to the low-ripple group (SPW-R power: *p* = 0.19; Ca^2+^: *p* < 0.01). Therefore, it was incorrect to assume that the two events of the SPW-R doublets were identical (Fig. 4a, left column). Because of this, the supralinearity of the summation was underestimated. The left column of Fig. 4a represents the worst-case scenario for the value of supralinearity. This is because the current, or any other mathematical approaches, separating the high- and low-ripple-associated Ca^2+^ responses would generate low-ripple-associated Ca^2+^ responses with a similar or smaller amplitude than the amplitude of the high-ripple-associated Ca^2+^ responses. Consequently, the sum of the two responses would be lower and, therefore, the supralinearity higher. To improve the calculation of summation, we replaced the second, high-ripple-associated Ca^2+^ responses in the left column of Fig. 4a with the average dendritic Ca^2+^ response from the low-ripple group and recalculated the supralinearity (Fig. 4a, right column). This second approach revealed an even larger supralinearity of dendritic summation during SPW-R doublets (compare “math. Σ low” to “doublet 2nd” in Fig. 4f–h and Supplementary Fig. 14b). Thus, we can conclude that the almost negligible low-ripple-associated Ca^2+^ signal can be increased to very high values when preceded by a high-ripple event during SPW-R doublets.

Next, we investigated whether the doublet-associated supralinear computation localized exclusively to the distal dendritic regions or is distributed more homogenously within individual PV+ neurons. Individual traces recorded along the entire somato-dendritic axis (Fig. 4b) and corresponding regional averages in individual neurons (Fig. 4c–h; Supplementary Figs. 12e and 14) and at the population level (Fig. 4f–h) showed that SPW-R-associated distal dendritic responses decreased rapidly below the distance threshold and, therefore, responses were an order of magnitude lower in the proximal dendritic and somatic regions. However, the relative amplitude of the low-ripple, high-ripple, doublet-associated responses, and the two mathematical sums, and therefore the amplitude of the SPW-R-associated supralinear summation, were preserved relative to the other responses along the somato-dendritic axis in individual neurons (Fig. 4c–e and Supplementary Fig. 14) and at the population level (Fig. 4f–h; *n* = 28/5/5 dendritic segments/cells/mice) indicating that the other computational rule associated with SWP-R doublets is not restricted to the distal dendritic compartments but is capable of propagating over the distance threshold to the soma, and effectively modulates the output of the neurons. The high efficiency of the output modulation of this effect is indicated by an over twofold amplitude increase (0.354, Δ*F*/*F*) during doublets relative to singlets (0.17, Δ*F*/*F*) in the proximal dendritic and somatic regions, which corresponds to about 14 APs according to the calibration curve (Supplementary Fig. 5c). In summary, our data indicated that the SPW-R-doublets and the associated dSpikes, which serve as an other layer of dendritic coincidence detection can effectively change the entire input-output transfer function of PV+ interneurons.

To test the underlying mechanism of dendritic coincidence detection during doublets, we reproduced SPW-R-dSpikes with two-photon glutamate uncaging of DNI-glutamate using spatially and temporally clustered input patterns (Supplementary Fig. 15a, b) as above. When two dSpikes were induced within a short temporal window (<100 ms), they induced large supralinear responses with decreasing amplitude as a function of time between the two events (Supplementary Fig. 15c, d). Dendritic spikes during in vivo SPW-R doublets showed a similar time-dependent decrease in their amplitude as a function of the inter-event time (Fig. 4j and Supplementary Fig. 12f), validating the length of the short temporal window of coincidence detection demonstrated with the uncaging measurements (Supplementary Fig. 15d). Similarly to singlets, doublet-associated dSpikes also showed a distance-dependent increase in their amplitude in vivo, but with a much steeper increase at the spatial threshold of the spike (Fig. 4b; Supplementary Figs. 12e and 14): this indicated an increased contribution of the non-linear dendritic mechanism in distal dendritic segments. Together, the supralinear integration of in vivo and in vitro dSpikes indicate that distal dendrites of PV+ cells can work as a coincidence detector for SPW-R-related neuronal assemblies by generating large, supralinear responses when the two SPW-R-associated assemblies are activated within a short temporal window and wire the same PV+ neuron.

## Discussion

While dendritic NMDA and Ca^2+^ spikes have been described in pyramidal cells^30,54–56^ and in some interneurons^34^, the existence of dSpikes in PV+ cells has remained controversial. Even though in vitro studies have demonstrated that dendrites of PV+ cells can generate supralinear integration when neuronal network activity is artificially increased or clustered photostimulation is implemented^36,57^, other in vitro reports have argued that dSpikes do not exist^58^; there has also been no in vivo validation^37^. However, previous in vivo measurements of PV+ cells have been restricted to short proximal dendritic segments located in a 2D plane and synchronized network activity has never been monitored locally: it is therefore impossible to detect SPW-dSpikes. To challenge the classical passive model of PV+ neurons under in vivo conditions in this study, we mapped regenerative events along the entire length of CA1 PV+ interneuron dendrites in 3D using fast 3D AO imaging through an eccentric deep-brain adapter: this enabled simultaneous LFP recordings from the ipsilateral hippocampus. This combination enabled, fast access to long, thin dendritic segments simultaneously in multiple locations along the entire 3D dendritic arbor of the PV+ cells (at a length of over 1500 µm) during locally detected SPW-R events in awake mice. We achieved, orders-of-magnitude higher speed and better signal-to-noise ratio than previous 2D imaging methods. Our approach revealed the existence of SPW-R-dSpikes in vivo. These were initiated in a minority of dendrites in distal dendritic regions from multiple hot spots and, following a time delay, they propagated towards the soma and to the more distal part of the dendrite. The amplitude of the SPW-R-dSpike decreased during propagation, with a pronounced drop at the critical spatial threshold (~150 µm from the soma).

The following criteria confirmed the regenerative nature of SPW-R-dSpikes: (i) the existence of a spatial threshold where the amplitude of the spike jumped to a higher value^59,60^, (ii) the time delay during initiation^34,36^, (iii) the fast propagation speed, which cannot be explained by Ca^2+^ diffusion^36,61^, (iv) the existence of a threshold in the number of uncaging-evoked synaptic inputs required to induce a dSpike^32,54^, and (v) the presence of NMDA and VGCC channel-based mechanisms in the central region and in the propagation zones, respectively^21,24,42^. These regenerative properties in our measurements were more robust under in vivo conditions than in previous in vitro measurements. As a noticeable example, the spatial distributions of the in vivo and in vitro SPW-R-dSpikes overlapped before the spatial threshold; however, under in vivo, but not in vitro, conditions there was a sharp amplitude increase above the spatial threshold, which resulted in a high and increasing magnitude of the SPW-R-dSpikes in the distal dendritic regions. This might be explained by the preserved, and functional, distal dendritic inputs arriving from the entorhinal cortex, which had been cut in acute slice.

NMDA receptor-dependent supralinear dendritic summation (~30%) has been reported earlier in PV+ cells^36,57^. These studies demonstrated similar input-output curves in oblique dendrites at relatively more proximal (~100 µm) locations, below the spatial threshold. In contrast to the limited capability of 2D laser scanning, the 3D AO technology was able to activate a 3-fold higher number of inputs and record more distal apical and basal dendritic segments, which were about 2–3 times thinner, about 4-fold longer, and were situated about 3-fold more distally from the soma (~200–300 µm). This method was, therefore, able to reach distal dendritic regions above the spatial threshold of dSpikes where dSpikes, following a sharp sigmoid-like increase at the threshold, had an order of magnitude higher amplitude than in the somatic/proximal dendritic regions, and where most of our results were discovered.

**Fig. 5:**
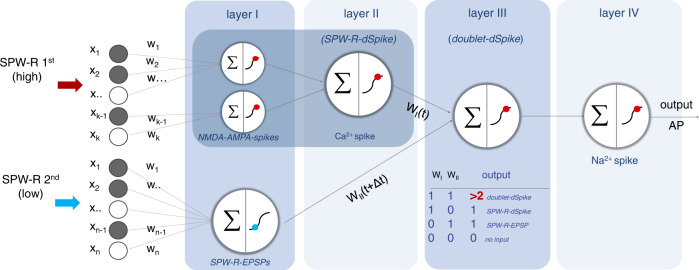
Schematic of dendritic non-linear computation in PV+ interneurons during SPW-Rs and SPW-R doublets. Our model comprises four supralinear layers, which covert information from dendritic inputs (*x*_i_) to the somatic output (output AP) during the two events of SPW-R-doublets (SPW-R 1st, SPW-R 2nd). Layer I: local dendritic activation above (NMDA-AMPA spikes) and below (SPW-R-EPSPs) the threshold of NMDA-AMPA spikes. Layer II: dendritic Ca^2+^ spikes (SPW-R-dSpikes), which appear on top of NMDA-AMPA spikes. Layer III: SPW-R doublet-associated dSpikes (doublet-dSpike). The inset in layer III shows a truth table for computation. Layer III works as a temporal coincidence detector: it is activated and generates large responses (doublet-dSpike) when both inputs (W_I_ and W_II_) are active (1, 1) with a time delay (Δ*t*) during SPW-R doublets. With all other input combinations [(1, 0), (0, 1), (0, 0)], it simply transmits the dendritic responses of layer I and layer II: the SPW-R-EPSPs and SPW-R-dSpikes.

Our results corroborate the recent modeling studies claiming that PV+ basket cells are two-stage integrators with non-linear dendritic arithmetic^39^. The two-stage model is based on sublinear and supralinear dendritic and linear somatic integrator units, and is also similar to the long-standing model of pyramidal cells^62^. In this study, we extend the two-stage model of PV+ cells by inserting another temporal integration stage between the dendritic and somatic integrators: the layer of SPW-R doublet-associated dSpikes (Fig. 5). The appearance of this supralinear dendritic integrator stage was exclusively associated with the temporal coincidence of two SPW-Rs, the events, which are one of the most synchronous network activities in the brain^5^ and, therefore, produce the highest amount of spatially and temporally synchronized inputs to dendrites, especially during SPW-R doublets. High-ripple-, low-ripple-, and doublet-associated signals dropped after the spatial threshold and had an order-of-magnitude smaller amplitude in the proximal dendritic and somatic regions; however, high-ripple- and doublet-associated signals were still able to induce about 7 and 14 APs, respectively, and the 2.5-3-fold relative increase for the supralinear summation during doublets was also preserved. This means that the results of the computational layer were effectively transmitted and preserved at the output of the cell. Moreover, based on our previous study^36^ and current results, we can divide the first, dendritic stage of the original two-stage model of PV cells into two layers: (i) the layer of NMDA-AMPA spikes, and (ii) the propagating Ca^2+^ spikes. A SPW-R-dSpike starts with an NMDA-AMPA spike, which is initiated at the first threshold in input number at dendritic hot spot regions and follows a sigmoid-like input–output curve. The propagating Ca^2+^ spike then appears after a short time delay at a higher, second threshold in input number^36^ on top of the NMDA-AMPA spike. In summary, we propose that PV interneurons can be characterized as four-stage integrators (Fig. 5) consisting of the following four layers: (1) NMDA-AMPA spikes, (2) propagating Ca^2+^ spikes, (3) a layer of SPW-R doublets, and (4) a somatic integration unit.

It has recently been shown that more cells were recruited when animals had to perform a more complex memory-demanding task, as this produces more complex SPW-R events (longer single events, or ripple bursts of doublets or even triplets) in about 20% of all cases^8–10^. The increased length of neuronal activity during these complex SPW-R events may reflect an enhanced integration process, when information originating from different brain regions (for example from the CA3 and MEC regions) is represented by different neuronal assemblies^10^, and integrated into the local CA1 network^9^. Supporting this, dendrites of PV+ interneurons in the CA1 receive inputs from the CA3 and MEC^63^ and are able to exhibit Hebbian and anti-Hebbian forms of synaptic plasticity, where the direction of plasticity is determined by the network state, the available Ca^2+^ sources, and the temporal and spatial clustering of the input pattern^35,38,64–67^. The spatiotemporally clustered dendritic inputs in pyramidal cells can induce supralinear voltage integration through NMDA receptors and voltage-gated Ca^2+^ and Na^+^ channels^28,30,54^: this is associated with a supralinear increase in dendritic intracellular Ca^2+^ concentration^28,34,54,68^. These non-linear dendritic Ca^2+^ signals can, in turn, activate biochemical cascades to induce synaptic or branch-specific short- or long-term plasticity: this may underlie memory formation^31,55,69–71^. Here, we demonstrated using fast 3D imaging, patterned photoactivation, and electrophysiological experiments that, similarly to pyramidal cells, PV+ cells could also generate increases in supralinear dendritic Ca^2+^ concentration with a very high amplitude: this happens when two SPW-R events (two SPW-R-related input assemblies) occur within a short time window (~150 ms) during SPW-R doublets. Therefore, PV+ dendrites could serve as a substrate for coincidence detection and neuronal plasticity during SPW-R events where neuronal network provides temporally clustered inputs to activate local supralinear dendritic computation. In addition to the temporal window, this form of dendritic integration also has a spatial window: the amplitude of the supralinearity and doublet-associated dSpikes showed a rapid amplitude rise at the critical spatial threshold (~150 µm). The SPW-R-dSpike, propagating centrifugally and centripetally through dendritic delay lines, can transfer this coincidence information to other distant inputs, where it might change the synaptic gains by generating plasticity.

In conclusion, our fast 3D imaging, photostimulation, and local electrophysiological recording methods demonstrate that SPW-Rs can induce dSpikes at distal parts of specific dendrites in hippocampal PV+ interneurons. These SPW-R-dSpikes are initiated from multiple hot spots, propagated centrifugally and centripetally: they also show a strong supralinear summation in distal dendrites above the distance threshold when two SPW-R events coincided within a short time window during doublets, and their local computational rulers were preserved at the output of the neurons. Therefore, we extended the currently available multi-stage integration model of PV+ neurons by inserting a temporal integration stage between the dendritic and somatic integrators: the layer of SPW-R doublets, which detects coincidence. Thus, PV+ interneurons not only transmit information but have a much more complex integration role in the hippocampal network that was previously associated only with pyramidal cells. From a computational point of view, we have not only discovered one more stage in neuronal signal integration, but also found that a short distal segment of a PV+ cell dendrite can provide a substrate for spatial and temporal coincidence detection of SPW-R-associated input assemblies, reflecting its potentially crucial role in the neuronal plasticity underlying hippocampus-dependent memory processes. However, further work is required to understand the causal link between supralinear dendritic integration and the direction and amplitude of plasticity and the fine-scale compartmentalization of the underlying biochemical mechanism in respect of the heterogeneity of the plasticity of PV+ cells.

## Methods

### Animals

All experiments were conducted in accordance with the Animal Care and Experimentation Committee of the Institute of Experimental Medicine (approval reference numbers PE/EA/1517-7/2018). PV-Cre adult mice (6–24 weeks old) of both sexes were used (*n* = 20 males, *n* = 15 females) and were housed in a temperature-controlled environment (24 ± 1 °C) on a 12 h reverse light cycle (dark period between 08:00 and 20:00) and humidity between 40 and 70%. The animals were kept in small groups (2–4 mice/homecage) in enriched environment with cardboard rolls, rotary discs, and extra nesting material (sizzle pet). During the training and the experimental period their water consumption was restricted to 1 ml/day after recovering from surgery. Weight loss due to water deprivation was kept below 20%. The mice had ad-libitum access to food. PV-GFP adult mice (6–24 weeks old) of both sexes were used (*n* = 11 males, *n* = 5 females) for in vitro electrophysiological and pharmacological experiments. PV-GFP animals have been described before^72^.

### Combined hippocampal in vivo imaging and LFP recordings

The hippocampal region of PV-Cre mice was injected with the pAAV.CAG.Flex.GCaMP6f.WPRE.SV40 plasmid (University of Pennsylvania Viral Vector Core) 4 to 6 weeks before the experiments. Mice were anesthetized with a fentanyl/midazolam cocktail (Richter/Egis, 0.1 ml/10 g bodyweight) and a small craniotomy (0.1 × 0.1 mm) was made to insert a bevelled injection needle (World Precision Instruments). We targeted PV interneurons in the dorsal hippocampal CA1 region (AP: −2.7 mm, ML: 2.1 mm, relative to bregma). Ropivacaine (Astra-Zeneca; 0.2%) was administered subcutaneously over the skull prior to the incision. Body temperature was maintained at 37 °C with a heating pad (TMP 5-b, Supertech). The needle was slowly lowered to a depth of 1200–1500 μm below the pial surface; the AAV virus (350 nl) was injected using a microsyringe pump (Micro4 microsyringe Pump Controller and Nanoliter 2010 Injector, World Precision Instruments). The needle was left in place for an additional 5–10 min to enable the AAV particles to diffuse. After removing the injection needle, the scalp was sutured, and animals were given a wake-up cocktail (Revertor: CP-Pharma GmbH, 2.5 mg/10 g bodyweight; Flumazenil: Fresenius Kabi GmbH, 2.5 mg/10 g bodyweight; Nexodal: G.L. Pharma GmbH, 1.2 mg/10 g bodyweight) to aid recovery. After surgery, saline solution (37 °C, 0.5 ml subcutaneous) was given to replenish the animals’ fluids. Finally, animals were treated with Melosolute (Cp-Pharma GmbH, 1 mg/10 g bodyweight) applied subcutaneously to minimize post-operative discomfort. Three weeks after the virus injection, the mice were placed on a heating pad (37 °C) and anesthetized deeply with a fentanyl/midazolam cocktail. Dexamethasone (Baxter Healthcare Corp., 0.2 mg/kg) and Baytril (Bayer, 200 µl/10 g bodyweight) were administered subcutaneously to reduce brain edema and local tissue inflammation. Ropivacaine (Astra-Zeneca, 0.2%, 0.05 ml) was applied subcutaneously over the skull prior to the surgery. An ophthalmic ointment was applied to the eyes to prevent drying during surgery. A custom-made stainless-steel head-plate was attached to the skull using dental cement (C&B Superbond) over the location of the virus injection for head-fixation (Supplementary Fig. 1). Subsequently, a 3 mm craniotomy was made above the hippocampus using a dental drill. The overlying cortex was carefully removed by aspiration (Aspiret, National Industrial Corporation) and replaced with a custom-made deep-brain adapter after sealing the vessels with a bipolar coagulator (Diatrom 80, Fazzini) and stopping the bleeding. The deep-brain adapter consisted of a silicon cylinder (diameter: ~3 mm, height: 1.2–1.4 mm) and a 4 mm diameter round coverslip (Glaswarenfabrik Karl Hecht GmbH & Co KG), which was eccentrically glued to the top of the cylinder with UV curable adhesive (Norland Products) to minimize the distance between the recording electrode and the cylinder while keeping the high mechanical stability during chronic recordings (Supplementary Fig. 1). The base of our custom-made silicone deep-brain adapter was made of silicone elastomer kit (Sylgard 184 silicone elastomer base and curing agent, Lot: 0008444444, Dow Corning Gmbh, USA), which contained two liquid components. When liquid components were thoroughly mixed manually with a spatula, the mixture (10 to 1 mix ratio) cured to a flexible elastomer. During mixing lots of bubbles were formed in the solution, therefore we waited about 30 min and the remaining bubbles were then removed with a needle. After that the liquid was kept at 125 C° until it solidified (25 min). Then we used a plunger to cut a 1.2-mm long cylinder with a diameter of 3 mm from the silicone band and we glued it eccentrically to a round coverslip with a diameter of 4 mm (defined as deep-brain adapter).

The spatial resolution (0.69 × 0.94 × 2.50 µm in the center) and diameter of the FOV were measured under the deep-brain adapter with fluorescent beads (diameter: 0.17 µm and 6 µm, respectively; Supplementary Fig. 2). To record LFP signals, a custom-made four-wire array (polyamide-imide coated 40 μm nichrome wires, Sandvik), with a distance between the recording sites of 100 or 200 μm, was implanted ipsilaterally into the dorsal hippocampus (AP: –2.5 mm, ML: 2.8 mm, relative to bregma) next to the cylinder at an angle of 80° under direct electrophysiological control to adjust to the proper depth. In this way, we minimized the distance between the electrode and the imaged neurons to maximize the possibility of detecting local SPW-R events. The four-wire electrode was then fixed to the skull with cyanoacrilate glue (Superbond, Loctite) and luting cement (3 M ESPE RelyX, 3 M). A simple pin electrode was implanted above the cortex on the contralateral site (AP: − 2.8 mm, ML: 2.8 mm, relative to bregma) and a reference electrode above the cerebellum through craniotomies made using a small drill-bit (0.3 mm diameter). The electrodes were connected to the amplifier board (RHD2132, Intan Technologies) with custom-made adapters. At the end of the surgery, the animals were given a wake-up cocktail (Revertor: CP-Pharma GmbH, 2.5 mg/10 g bodyweight; Flumazenil: Fresenius Kabi GmbH, 2.5 mg/10 g bodyweight; Nexodal, G.L. Pharma GmbH, 1.2 mg/10 g bodyweight) to aid recovery and were treated with Ringer’s lactate solution and Melosolute for the next 3 days (CP-Pharma GmbH, 1 mg/10 g bodyweight) to minimize post-operative discomfort. Baytril (Bayer, 2.5%, 200 μl/10 g bodyweight) was administered to reduce tissue inflammation.

### Analysis of electrophysiological data, detection of SPW-R events

Local LFP signals were recorded using the RHD2132 amplifier board (Intan Technologies, version 1.41) with an amplifier bandwidth from 0.1 Hz to 7.5 kHz and sampled at 20 kHz. Data were analyzed and SPW-Rs were then detected using MATLAB-based software (MES5 version 2043-MES6 version 11391, Femtonics). In order to preserve the phase and amplitude of individual ripple cycles in the LFP signal, we used the difference between two low-pass filters as previously described^36^. To eliminate unit activity, we set the two cut-off frequencies of the two Gaussian filters to 150 Hz and 500 Hz. Only the channel with the highest ripple band LFP power, and events exceeding 4 SD over the baseline were analyzed. Signals were discarded when they were also present on the contralateral cortical electrode. The electrophysiological and the simultaneously recorded Ca^2+^ imaging data were overlaid and analyzed in a MATLAB-based program (MES, Femtonics) with a delay between ripple peak and Ca^2+^ peak of 29.02 ± 4.73 ms.

### Anatomy and immunohistochemistry

After completing the in vivo experiments, mice were deeply anesthetized with isoflurane and perfused transcardially with saline followed by ice-cold 4% paraformaldehyde. Brains were removed and post-fixed for 24 h, cryoprotected in 10% sucrose/PBS, and sectioned at 30 µm thickness on a sledge microtome. After defatting, sections were stained for 5 min in cresyl violet solution (C5042, Sigma-Aldrich) then dehydrated and coverslipped with Permount (Thermo Fisher Scientific) to examine the degree of tissue damage induced by inserting the eccentric deep-brain adapter (Supplementary Fig. 1). To assess the colocalization of PV+ interneurons with GCaMP6f signal in the brain parenchyma, free-floating sections were incubated overnight in rabbit anti-PV27 primary antibody (Swant, Switzerland, Lot No. 2014, 1:1000, RRID: AB 2631173) in the presence of 0.3% Triton X-100, followed by adequate fluorochrome-conjugated secondary antibody (Lifetech, A21207, donkey anti-rabbit Alexa 594 1:500, RRID: AB 141637). Images were captured using an Axiovert 200 M microscope (Zeiss) using Axiovision 4.8 software.

### Habituation of water-deprived animals

The handling period started 1 week after implantation of the head-plate: mice were habituated to the two-photon microscope in the head-fixed position for 1–3 weeks before the start of the experiments. To increase the probability of detecting SPW-Rs, we used water-restricted mice and provided them with a small amount of water (~5 µl per trial) during imaging sessions through a silicon/plastic tube. Water rewards were delivered using a custom-designed Bpod device^73^.

### In vitro electrophysiology and pharmacology

Acute, 300 μm thick coronal hippocampal slices were prepared for in vitro pharmacological experiments. We used 40- to 60-day-old transgenic mice expressing enhanced green fluorescent protein (eGFP) controlled by the parvalbumin promoter^36,53,72^, or PVA/IRES-cre mice injected with a pAAV.CAG.Flex.GCaMP6f.WPRE.SV40 plasmid (University of Pennsylvania Viral Vector Core, RRID: Addgene 100835) 4–6 weeks before the experiments. During the procedure, animals were deeply anesthetized by isoflurane inhalation and then suddenly decapitated. The brain was removed and put into an ice-cold cutting solution containing (in mM): 2.8 KCl, 1 MgCl_2_, 2 MgSO_4_, 1.25 NaH_2_PO_4_, 1 CaCl_2_, 10 D-glucose, 26 NaHCO_3_ and 206 sucrose^36,53^. The brain slices were cut using a vibratome (VT1000S, Leica) and stored at room temperature (23–25 °C) in standard artificial cerebrospinal fluid containing (in mM): 126 NaCl, 2.5 KCl, 2 CaCl_2_, 2 MgCl_2_, 1.25 NaH_2_PO_4_, 26 NaHCO_3_ and 10 glucose, and bubbled with carbogen gas^52,53^. We used a regular submerged chamber with normal perfusion rate. PV+ interneurons in the CA1 stratum oriens layer were visualized for targeted patch-clamp recordings by infrared oblique illumination (at 880 nm, Femtonics) and two-photon imaging (830 or 900 nm). Whole-cell current-clamp recordings (MultiClamp 700B, Digidata 1440: Molecular Devices) were taken at 32–34 °C (in-line heater: Supertech; chamber heater: Luigs & Neumann). Data were recorded using pClamp10 (Molecular Devices) and MES (Femtonics) software. Borosilicate glass electrodes (6–9 MΩ resistance) filled with an intracellular solution containing (in mM): 125 K-gluconate, 20 KCl, 10 HEPES, 10 di-tris-salt phosphocreatine, 0.3 Na-GTP, 4 Mg-ATP, 10 NaCl and 0.008 biocytin were used for whole-cell recordings. For the pharmacology patch, pipettes also contained 100 μM Fluo-4 (Invitrogen) combined with 100 μM Alexa 594 (Invitrogen). For experiments with GCaMP6f labeling, patch pipettes contained the same intracellular solution but without Fluo-4. Tetrodotoxin (1 μM), nimodipine (20 μM), mibefradil (10 μM), ω-conotoxin MVIIC (0.5 μM), 6-cyano-2,3dihydroxy-7-nitro-quinoxaline (CNQX) (10 μM), and D,L-2-amino-5phosphonopentanoic acid (AP5) (60 μM) were purchased from Tocris. The cocktail of voltage-gated calcium-channel (VGCC) blockers also contained ω-conotoxin MVIIC, nimodipine, and mibefradil. All drugs were applied in the bath.

### Three-dimensional two-photon imaging

All two-photon experiments were performed with a 3D AO laser-scanning microscope (ATLAS, Femtonics), which upgraded a standard two-photon system (Femto2D-Dual, Femtonics) through the camera port to a fast, 3D laser-scanning microscope. Femtosecond laser pulses were provided by a Mai Tai HP laser (Spectra Physics) and by a Chameleon Ultra II laser (Coherent) operated at 910 nm wavelength for GCaMP6f measurements: the pulse length was set to 100 fs below the objective using the motorized four-prism sequence of the ATLAS microscope (4DBCU unit, Femtonics). We increased the total transmission of the 3D AO microscope by 77% (from 14.3 ± 4.1% to 25.5 ± 6.0%, recorded at the objective) compared to the previous version^46^, which increased the fast x-, y-, and z-scanning ranges of the microscope. We used a 16× water-immersion objective lens with 0.8 NA and 3 mm WD (Nikon CFI LWD Plan Fluorite Objective, N16XLWD-PF). Multiple long dendrites of the PV+ cells were simultaneously recorded using fast 3D ribbon scanning at 63.94 ± 18.43 Hz, using the 3D AO scan head^46^. The network activity of PV+ cells was recorded using either the fast arbitrary frame-scanning mode of the ATLAS microscope or the resonant scan head of the Femto2D-Dual microscope.

Two-photon measurements above a certain intensity threshold can induce photodamage in dendrites, which can be recorded in different parameters, for example, as a sudden or modest drop in signal amplitude, or an increase in the dendritic diameter (dendritic swelling). Therefore, to test a potential contribution of phototoxicity to our measurements, we recorded anatomical and physiological parameters such as dendrite diameter, baseline fluorescence, response amplitude, response full width at half maximum, decay time, and rise time during long-term recordings (Supplementary Fig. 3). None of these parameters showed any significant change as a function of time in a 41-min recording period: this confirms that there are no phototoxicity effects in our measurements (Supplementary Fig. 3). Low phototoxicity is one of the main advantages of 3D AO scanning: this method inherently incorporates an optimal measurement strategy, which is also defined as low-power temporal oversampling (LOTOS)^50^. Briefly, if the same signal-to-noise ratio of the Ca^2+^ transients is realized by scanning the same location at a higher frequency, then the lower peak intensity is sufficient to assure the same signal-to-noise ratio, which reduces phototoxicity.

### Fast arbitrary frame-scanning mode for 3D in vivo imaging

Three-dimensional AO scanning can provide over 10^6^× faster scanning speed or over 10^3^× higher signal-to-noise ratio than classical raster scanning of the same volume (see Equation S84 in ref. 46) because it records only the preselected regions of interests (ROI) and skips the unnecessary areas: however, the maximal length of 3D lines, which can be generated from any point in any desired direction in 3D within a single AO cycle (20–33 µs, defined as 3D AO drift) was limited to about 100–150 µm^46^. This also limited the maximal diameter of the field-of-view (and the scanning volume in fast volumetric imaging), which can be composed of a matrix of single 3D AO drifts. To get larger fields-of-view from single 3D drifts, we optimized command frequencies, keeping the frequency changes relatively small on the first pair of crystals. This is because the length of the 3D drifts, and hence the diameter of the field of view, is dominantly limited by the tolerance for the incoming light angle on the second pair of crystals. We therefore used the x and y deflectors simultaneously to scan in a 45° rotated horizontal plane and added –200 µm z-focusing with the AO deflectors to minimize variance in incident angle during scanning, and to maximize the use of bandwidth at the second group of AO deflectors. This process increased the maximal length of the 3D AO drifts and, hence, the maximal diameter of the field-of-view to 500 µm with the 20× Olympus objective (XLUMPLFLN-W 20×, NA = 1.0), which is close to the theoretical maximum (700 µm). We were therefore able to image 500 × 500 µm frames with 510 × 510-pixel resolution and ~50 Hz speed, outperforming the ~30 Hz frame rate of standard resonant-galvo-based multiphoton microscopes. Moreover, as these high-speed frames are composed of 3D AO drifts, we were able to arbitrarily rotate them in the entire 3D scanning volume. This enabled us to visualize the rich dendritic arborization of PV+ cells more easily, by properly matching their imaging planes. Therefore, different ROI scanning modes such as chessboard and 3D ribbon scanning can be enhanced by various tilted ROI shapes. In summary, we solved a major limitation of fast 3D AO scanning by increasing the imaging speed of entire planes and contiguous volumes.

### Analysis of imaging data

Δ*F/F* was calculated using the built-in analysis tools in the acquisition software (MES5 and 6, Femtonics). Raw fluorescent data (*F*) recorded along ribbons in 3D were spatially normalized, and then projected onto a 2D plot as a function of longitudinal (*d*_LONG_) and transverse (*d*_TRANSV_) distance along the scanning ribbons: *F(d*_LONG_, *d*_TRANSV_*)*. Relative fluorescence change was calculated as:

1 $$\varDelta F/F\,=\,\frac{(F({d}_{{{{{{{\mathrm{LONG}}}}}}}},\,{d}_{{{{{{{\mathrm{TRANSV}}}}}}}},\,t)\,{\mbox{--}}\,{F}_{0}({d}_{{{{{{{\mathrm{LONG}}}}}}}},\,{d}_{{{{{{{\mathrm{TRANSV}}}}}}}}))}{{F}_{0}({d}_{{{{{{{\mathrm{LONG}}}}}}}},\,{d}_{{{{{{{\mathrm{TRANSV}}}}}}}})\,}$$

where *t* denotes time and *F*_0_ is the baseline fluorescence level. We used the ImageJ open-source software with some custom-written macros https://github.com/SJLinda/Imagejmacro and the MES5 and 6 analysis program (Femtonics) to generate projections from the data recorded in 3D. The analysis and measurement processes were similar to those described previously^34,46^. We considered a neuron in a given trial as active if the difference between the peak Δ*F*/*F* value of the baseline epoch was higher than 2 standard deviations (SD). The peak Δ*F*/*F* value was defined here as the average Δ*F*/*F* value of the datapoints around the peak in the range of 60 ms.

The 3D ribbon scanning frames recorded not only the preselected dendrites but also the surrounding background areas, enabling us to monitor and eliminate neuropil contamination from our responses (Supplementary Fig. 6). Our data confirmed that neuropil responses did not contribute to the distance-dependent increase in the SPW-R-dSpikes (Supplementary Fig. 6c–e). The ribbons selected for 3D recordings in our measurements typically contained multiple dendrites and axons crossing the selected dendritic segment of interest (e.g., Supplementary Fig. 6b). The images generated by 3D ribbons enabled to monitor and avoid these subregions with crossing neuropil during analysis (e.g., Supplementary Fig. 6c). In summary, our methods allowed us to monitor and avoid contamination from other structures (e.g., neuropil, background) during the readout of the signal (∆F/F) from the selected PV+ dendritic segment.

To test whether the non-linearity of the GCaMP6f sensor contributed to our supralinear responses, we used somatic current injections to induce AP potential bursts, and plotted the amplitude of dendritic responses as a function of the number of APs (Supplementary Fig. 5). Our data indicated that the GCaMP6f sensor in itself did not contribute to the large supralinearity of the dendritic responses during SPW-R-dSpikes.

We used a motion-correction algorithm as described earlier^46^. If motion was relevant during a scanning cycle, we performed non-rigid motion correction using the open-source software package elastic, adjusting its hyperparameters to our two-photon specific image properties^74,75^. As well as using the NoRMCorre algorithm for weak distortion, we also used the elastic software package with two-photon specific parameters when large, non-homogeneous transformations were needed^76^.

### In vitro two-photon uncaging

Simultaneous glutamate uncaging and imaging experiments were performed as previously described^36^. DNI-Glu•TFA (Femtonics, 2.5 mM) was applied in the ACSF bath^36^. Glutamate was released using a second ultrafast pulsed laser (Mai Tai, SpectraPhysics) at 740 nm wavelength according to the red-shifted two-photon excitation spectrum of the caged compound. The laser light was coupled to the two-photon microscope using a polarized cube (PBS10; Thorlabs) and two motorized mirrors to precisely overlap the uncaging and imaging laser paths. Fast imaging of long dendritic segments was interleaved during the uncaging periods when the uncaging laser beam jumped to the preselected locations. The laser intensity at the focal spot of uncaging was set to induce EPSPs with an amplitude similar to the amplitude of unitary EPSPs evoked by local puffed high osmolar ACSF^34,36^. Many unitary inputs (15-15 at each side with 1 μm spacing) were activated in a short time period to evoke dSpikes similar to the SPW-R-dSpikes.

### Data analysis and statistics

As a first step towards separating SPW-Rs and the simultaneously recorded dendritic responses into multiple functional subpopulations, we selected three standard SPW-R parameters and generated the corresponding spectral histograms: the area of Ca^2+^ responses, the peak-to-peak amplitude, and the duration of the ripples and generated the corresponding spectral histograms (Fig. 3a). Both the spectral histogram of area after logarithmic transformation and the duration of the ripples, were analyzed using the Peak Analyzer module (Origin Pro, OriginLab), which detected two peaks with Gaussian fitting in both spectral histograms (Fig. 3a). This separated SPW-Rs and the associated dendritic responses into three groups: the low-ripple group, the high-ripple group, and the group of SPW-R doublets (Fig. 3a). The spectral histogram of the peak-to-peak amplitudes reinforced the separation of the three groups (Fig. 3a). The existence of the group of doublets has already been published with standard methods. Therefore, we introduced a gap statistic method to further validate the separation of these two groups. We sorted the events as a function of the log(Ca^2+^ area), which revealed a gap, in the form of a jump-like increase in the amplitude, which separated low-ripples from high-ripples and doublets. To determine the statistical significance of the gap, we calculated the average (mean ± SD) distance to the six closest neighbors (Fig. 3c, bottom) and found a significant increase in the distance at the gap. The *p*-values of the Student’s *t-*test on the center four points compared to the baseline values were: 0.00134, 2.38 × 10^−7^, 3.08 × 10^−6^, and 0.00011 when assuming equal variance (Fig. 3c). When equal variance is not assumed (Welch correction), the same *p*-values were: 0.150, 0.024, 0.040, and 0.147, indicating a significant increase in the distances at the gap with both approaches. These data reinforced the separation of the low-ripple group from the two other groups.

Two methods were used for cluster analysis. *K*-means cluster analysis of the SPW-Rs and the simultaneously recorded dendritic responses separated doublets from singlets but kept the high- and low-ripples, in one single group. However, a second *K*-means cluster analysis performed only on singlets was able to reproduce the high- and low-ripple groups shown in Fig. 3a with only 3 outliers. In summary, *K*-means cluster analysis was able to reproduce the three groups of SPW-R-associated events identified with spectral histograms but only when applied in two steps. As a one-step alternative to *K*-means cluster analysis, we used t-distributed stochastic neighbor embedding (tSNE) on 817 datapoints from 32 dendritic segments, trying to minimize the Kullback–Leibler divergence between the different distributions the data came from. We found that clusters in tSNE spaces fit to the original group with only a small number of misclassifications. tSNE constructs a set of embedded points in a low-dimensional space whose relative similarities mimic those of the original high-dimensional points. The embedded points show the clustering in the original data. Roughly, the algorithm models the original points as coming from a Gaussian distribution, and the embedded points as coming from a Student’s *t-*distribution (Supplementary Fig. 10a).

To investigate whether the signal-to-noise ratio of the dendritic measurement could affect the separation of the three groups of SPW-R-associated responses we calculated the average SD of the fast 3D dendritic measurements (SD_avr_), which was 4.61 ± 0.43% (∆*F*/*F*, *n* = 10). Data below SD and 2×SD were below the gap separating the low-ripples from the high-ripples and doublets (Fig. 3c), indicating that signal-to-noise ratio did not affect the separation.

To further validate the existence of the three separate groups we introduced a distance metric based on the product of six SPW-R parameters: this separated the same three groups of responses when combined with cluster analysis (Supplemental Methods, Supplementary Fig. 11).

### Identification of dendritic Ca^2+^ transients

Three-dimensional imaging revealed two major types of dendritic activation. One of them was linked to SPW-R-associated events: activity was typically restricted to one dendritic segment with a high response amplitude in the distal region (local activation mode; Fig. 1i). The other occurred between SPW-R events and was represented by a global activation in all dendritic segments, with a high amplitude in the somatic and proximal dendritic domains (global activation mode, Fig. 1j). The global and local activation modes formed two antagonistic populations according to the spatial distribution of the associated dendritic responses: distal dendritic responses were large and decreased as a function of distance toward the somatic region during the local activation mode (Fig. 2a–d; Supplementary Fig. 4 and Supplementary Movie 2). In contrast, responses during the global activation mode showed an antagonistic distribution: the large somatic responses decreased as a function of distance from the soma (Fig. 2a–d and Supplementary Fig. 4). The spatial distributions of backpropagating action potentials (bAPs) and SPW-R-associated dendritic spikes has shown the same antagonistic relationship (bAPs decreased and the dendritic spikes increased as a function of distance from the soma) under in vitro conditions^36^ as the global and local in vivo activation modes in this study (Supplementary Fig. 4). Moreover, the spatial distribution profile of these in vivo and in vitro events overlapped pairwise: in vitro SPW-R-associated dendritic spikes and bAPs were similar to the local and global activation modes, respectively (Supplementary Fig. 4). Their transients also had similar kinetics under in vivo and in vitro conditions (decay time constants for in vitro bAPs: 356.23 ± 3.63 ms, *n* = 12; for in vivo bAPs: 339.67 ± 26.79 ms, *n* = 15; *t*-test, *p* = 0.5. Therefore, we identified the local and global activation modes as SPW-R-associated dendritic Ca^2+^ spikes (SPW-R-dSpike) and bAPs, respectively.

### Identification of SPW-R-dSpikes

Local LFP signals were recorded using an Intan RHD2132 interface board (Intan Technologies) with an amplifier bandwidth from 0.1 Hz to 7.5 kHz and sampled at 20 kHz. Data were analyzed and SPW-Rs were then detected using MATLAB-based software (MES5 and 6, Femtonics). In order to preserve the phase and amplitude of individual ripple cycles in the LFP signal, we used the difference between two low-pass filters, as previously described^36^. To eliminate unit activity, we set the two cut-off frequencies to 150 Hz and 500 Hz and used the difference between the two Gaussian filters. Only the channel with the highest ripple band LFP power, and events exceeding 4 SD over the baseline were analyzed. Signals were discarded if they were also present on the contralateral cortical electrode. The start (*T*_1_) and end (*T*_2_) points of the ripple envelope were defined as the first time points when the LFP power exceeded the baseline by 4 SD, and when the power returned below 4 SD, respectively. The ripple area and ripple power were defined as the integral of the absolute value and the square of the LFP signal between *T*_1_ and *T*_2_, respectively. The ripple frequency was defined as the number of individual ripple cycles between *T*_1_ and *T*_2_ divided by *T*_2_ − *T*_1_. The peak-to-peak value of the ripple event was defined as the difference between the maximum and minimum value of the LFP signal within the [*T*_1_, *T*_2_] interval. The ripple duration (width) was defined as *T*_2_ − *T*_1_ when only one SPW-R event occurred within a 300 ms interval. If two events were generated, then the start (*T*_21_) and end (*T*_22_) points of the envelope of the second ripple event were defined as the first time points when the LFP power of the second event exceeded the baseline by 4 SD and when the power returned below 4 SD, respectively. The duration of the entire complex was defined in this case as *T*_22_ – *T*_1_ and LFP activity was accepted as one SPW-R complex if *T*_22 _– *T*_1_ < 300 ms. This definition resulted in two separate peaks on frequency histograms of the SPW-R duration (Fig. 3a), which were defined as SPW-R singlets and doublets and had a mean duration of 35.6 ± 1.44 ms and 181.62 ± 12.165 ms, respectively (mean ± SEM).

We considered the somatic or a dendritic region of a neuron as active in a given trial if the difference between the peak Δ*F*/*F* value of the baseline epoch was higher than 2 SD. The SD was calculated from the first 100–500 ms time interval of the individual measurement trials. The start (*T*_3_) and stop (*T*_4_) points of the Ca^2+^ curve was defined as the time when the Ca^2+^ response crossed the 2 SD threshold over the baseline. The area and maximum of the Ca^2+^ responses were calculated as the integrals in the [*T*_3_, *T*_4_] interval, and in a 60 ms interval centered to the peak, respectively. The maximum of the absolute value of the individual SPW-R events was defined as time zero. The LFP signals and the associated Ca^2+^ responses were simultaneously shifted to time zero before the correlative analysis to preserve the original time delay. A Ca^2+^ response and an SPW-R event were defined as simultaneous when the [*T*_3_, *T*_peak_] and [*T*_1_, *T*_2_] intervals overlapped, where *T*_peak_ means the time of the peak of the Ca^2+^ response. This definition resulted in a jitter of 29.02 ± 4.73 ms (mean ± SEM) between the peak of the SPW-R events and the peak of the Ca^2+^ responses. 85 of the 307 SPW-R events detected were associated with a propagating dSpike: this corresponds to a 27.6% total activation ratio. 12 of the recorded 58 dendrites were active, corresponding to an activation ratio of 21%. The average activation ratio of dSpikes (dSpikes/SPW-R events) for individual neurons was 29.9 ± 4.1% (mean ± SEM, *n* = 12 cells, *n* = 6 mice).

### Statistics and reproducibility

All values were reported as mean and error bars as ±SEM. Box-and-whisker plots show the median, 25th and 75th percentiles, range of nonoutliers and outliers. Statistical significance was tested using a one way or two-way Student’s paired or unpaired *t*-test. Both the spectral histogram of data after logarithmic transformation and the duration of the ripples, were analyzed using the Peak Analyzer module (Origin Pro, OriginLab), which detected two peaks with Gaussian fitting in both spectral histograms. We introduced a gap statistic method to further validate the separation of the two ripple groups. Two methods were used for cluster analysis. *K*-means cluster analysis of the SPW-Rs and the simultaneously recorded dendritic responses separated doublets from singlets but kept the high- and low-ripples, in one single group. As a one-step alternative to *K*-means cluster analysis, we used *t*-distributed stochastic neighbor embedding (tSNE), trying to minimize the Kullback–Leibler divergence between the different distributions the data came from. Confocal and two-photon images used as representation were replicated successfully for over different animals, with identical results. Calculations were performed in Microsoft Excel, Origin Pro, OriginLab. Statistical differences with *p* < 0.05 were considered significant. In figures, a single asterisk (*), double asterisks (**), and triple asterisks (***) indicate *p* < 0.05, *p* < 0.01, and *p* < 0.001, respectively, and are used throughout the manuscript.

### Reporting summary

Further information on research design is available in the Nature Portfolio Reporting Summary linked to this article.

## Supplementary information

Supplementary Information

Description of Additional Supplementary Files

Supplementary Movie 1

Supplementary Movie 2

Reporting Summary

## Data Availability

Additional image data files are available from the corresponding author (rozsabal@koki.hu) upon reasonable request. Source data are provided with this paper.

## Source data

Source Data

## Code availability

We used the ImageJ custom-written macros: https://github.com/SJLinda/Imagejmacro. Additional analysis routines and software are available from the corresponding author (rozsabal@koki.hu) upon reasonable request. Bpod and Pulse Pal devices are available from Adam Kepecs (akepecs@wustl.edu).
