# Supplementary material for: Sharp-wave ripple doublets induce complex dendritic spikes in parvalbumin interneurons in vivo
Source: Nat Commun. 2022 Nov 7;13:6715. doi: 10.1038/s41467-022-34520-1 (PMC9640570; doi:10.1038/s41467-022-34520-1)
Supplement: Supplementary file 1 — Supplementary Information [file 41467_2022_34520_MOESM1_ESM.pdf]

Supplementary Figures

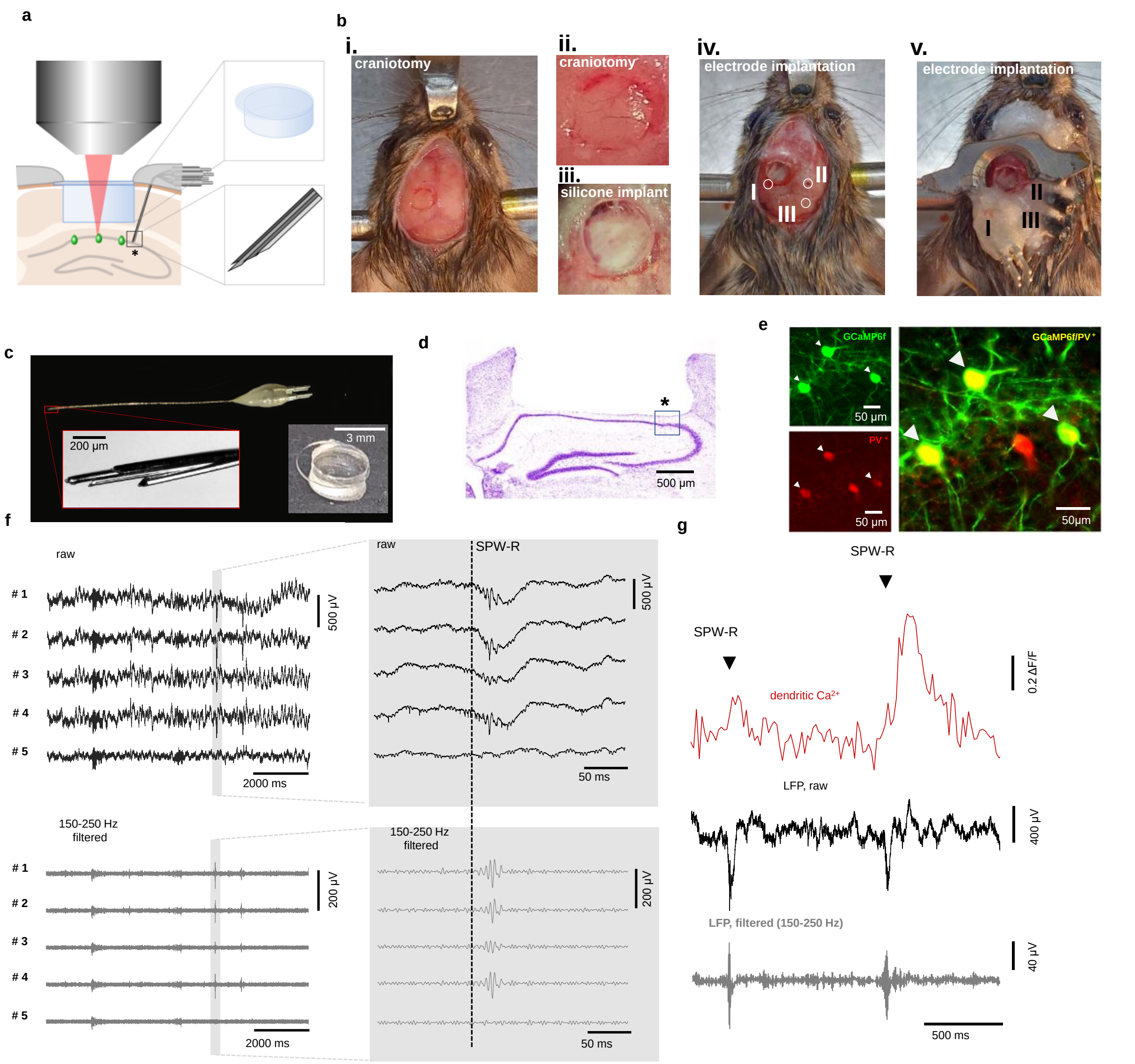

Supplementary Figure 1, Simultaneous 3D imaging and multi-channel recording of the local LFP from the hippocampus using an eccentric deep-brain image adapter. a, Schematic of the experimental design. Fast 3D imaging was performed in the CA1 region of the hippocampus through an eccentric deep-brain adapter which enabled large scanning volumes and the simultaneous use of a custom-made flexible four-wire electrode for local LFP detection in close proximity to the imaged PV+ cells (black asterisk). The contralateral cortical and the reference electrodes are not shown. **b,** Cranial window with the head holder, the recording electrodes, and the eccentric deep-brain adapter. (i-ii) Images show the craniotomy during surgery. (iii) Image of the eccentric deep-brain adapter used as a plug to replace the removed cortex. (iv-v) Images show the location of the hippocampal (I), the contralateral cortical (II), and the reference electrode (III) which was above the cerebellum. **c,** Image of a custom-made four-wire electrode used for multi-channel LFP recordings. **d,** Coronal section of the fixed, Nissl-stained brain after in vivo imaging. The location of the hippocampal electrode (I) is indicated by a black box and asterisk at the posterior edge of the cranial window. The reference (II) and the cortical (III) electrodes are not visible in the plane of the section. **e,**

Immunohistological validation of the GCaMP6f labelling of the PV+ neurons in the hippocampus. Confocal image of the PV+ interneurons labelled with rabbit anti-PV27 primary antibody. The arrowheads indicate co-labelling: PV+ interneurons expressing GCaMP6f. **f,** Multi-channel recording of LFP signal was performed simultaneously with fast 3D dendritic imaging. Exemplified traces for local hippocampal (channel #1–#4) and contralateral cortical (channel #5) LFP recordings (black: raw, grey: filtered data). Grey box indicates enlarged view. Dashed line indicates initiation of an SPW-R event. In previously published combined measurements, the LFP signal was recorded from the contralateral hippocampus: this enabled investigation of only the bilateral SPW-R events[1–6]. **g,** Example of the simultaneous LFP and fast 3D dendritic recording with two successive SPW-R events (arrowheads). Note the large variability at consecutive events.

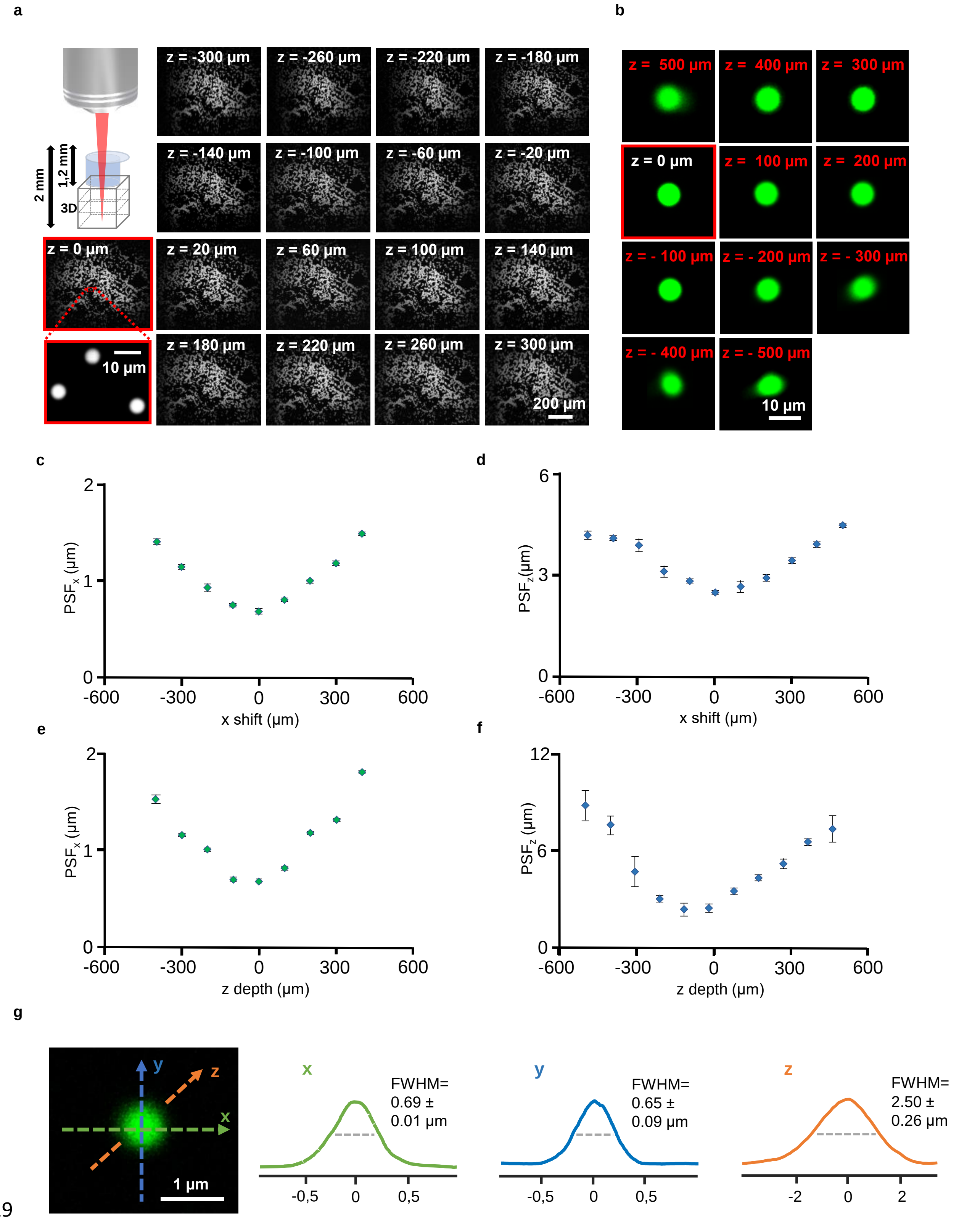

Supplementary Figure 2, Characterization of the field-of-view and resolution during fast 3D AO imaging through the eccentric deep-brain image adapter. a, Top left, schematic of the measurement. A fluorescent sample consisting of 6 µm-diameter beads was imaged with 3D AO imaging at 910 nm through the eccentric deep-brain image adapter using the 16× objective (0.8 NA, Nikon). Images show the maximal field of view with 3D AO imaging through the

adapter at different fast AO z-focusing levels (from −300 µm to +300 µm). Numbers show the z shifts required to compensate for the different AO z-focusing levels, with the mechanical arm and the step motor to keep the beads in focus. The maximal field of view was about 600 µm at z=0 µm. **b,** Image of a 6 µm-diameter bead at 910 nm at different AO z-focusing levels (from −500 µm to +500 µm, fast z range = 1000 µm). Red numbers indicate the mechanical z shift required to keep the bead in focus. Note the high spatial resolution preserved in the entire 1000 µm fast AO z-scanning range. The z projection of the images consists of 82+18 planes on average. **c-f,** Resolution was measured on small fluorescence beads (diameter: 170 nm) at 910 nm and plotted as a function of distance along the x and z axes and determined as the full width at half maximum values (mean ± SEM, n=96 recordings, n=4 beads) of the point-spread function along the x and z axes. The high spatial resolution characterizing two-photon excitation through a long working distance objective was preserved in the entire scanning range through the eccentric deep-brain image adapter during fast 3D recordings. In addition, the high resolution required to image fine details of the neuropil (such as dendritic spines or the very thin dendrites of PV cells) was also preserved in the central volume of about 400 × 400 × 400 μm^3. **g,** Average normalized fluorescence histograms along the x, y and z axes (n=4 measurements with n=4 beads) of a 170-nm fluorescence bead. Resolution was determined as the full width at half maximum (FWHM) in every histogram (mean ± SEM).

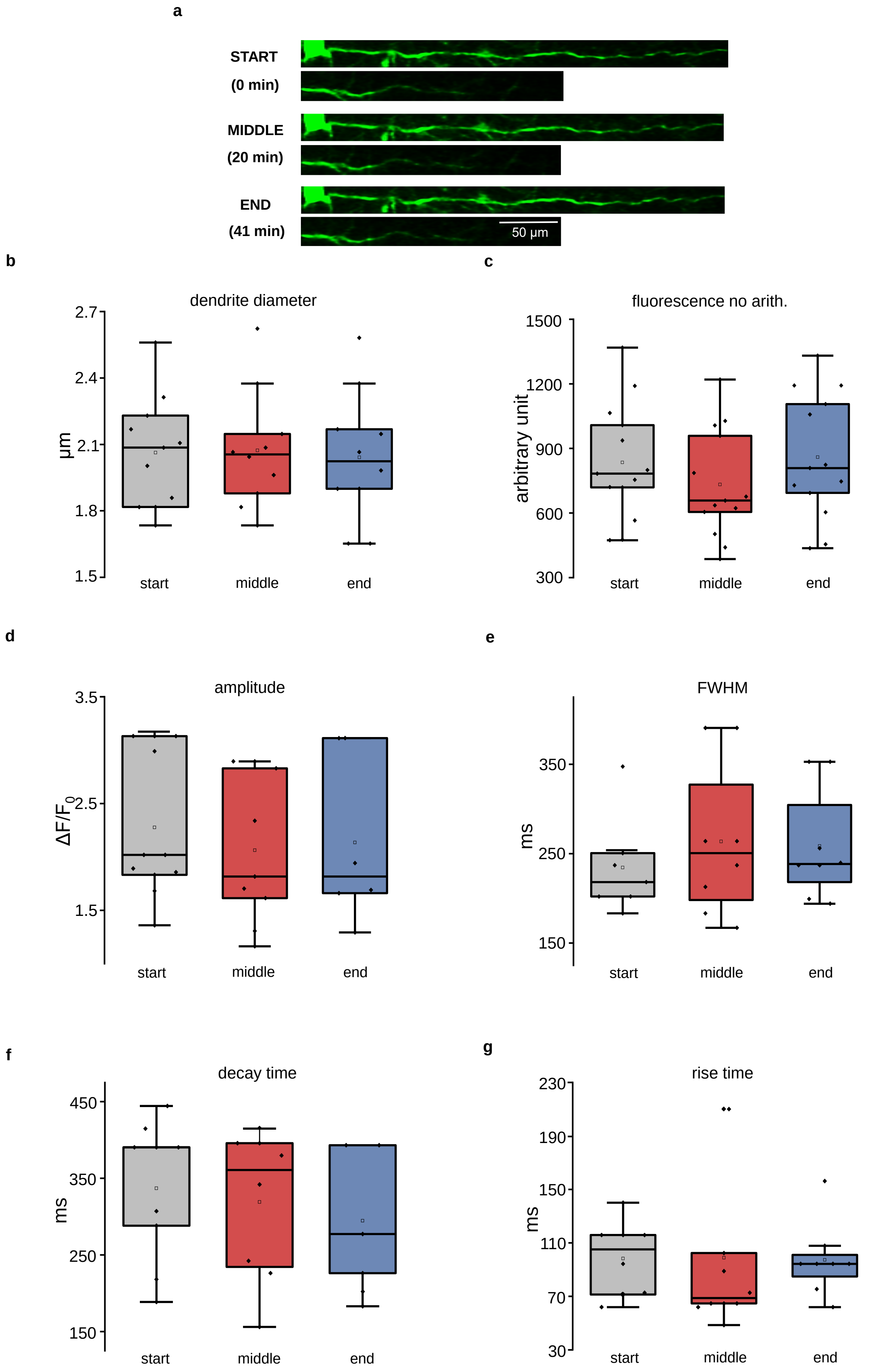
a
START
(0 min)
MIDDLE
(20 min)
END
(41 min)
50 µm
b
dendrite diameter
µm
2.7
2.4
2.1
1.8
1.5
start
middle
end
c
fluorescence no arith.
arbitrary unit
1500
1200
900
600
300
start
middle
end
d
amplitude
ΔF/F0
3.5
2.5
1.5
start
middle
end
e
FWHM
ms
350
250
150
start
middle
end
f
decay time
ms
450
350
250
150
start
middle
end
g
rise time
ms
230
190
150
110
70
30
start
middle
end

Supplementary Figure 3, **The physiological and anatomical parameters of dendritic segments of PV cell did not change as function of time during long-term measurements**. **a**, 2D projection images of a long dendritic segment of a GCaMP6f-labelled PV+ cells during fast 3D ribbon scanning at the beginning (0 min), middle (20 mins) and end (41 mins) of the measurements. Note that dendritic and somatic morphology do not change during long recordings. The projection of this representative dendrites consists of 595 frames and measured over 10 seconds. **b-g**, Quantitative analysis of the long-term stability of the anatomical and physiological parameters of the measured neurons: dendrite diameter (b), baseline fluorescence (n=31 measurements) (c), SPW-R-dSpike response amplitude (n=38 measurements) (d), full width at half maximum (FWHM, n=26 measurements) e), decay time (n=23 measurements) (f), and rise time (n=23 measurements), (g) at the beginning (0 min), middle (20 mins) and end (41 mins) of the n=26 measurements. The presented parameters did not show any significant change as a function of measurement time, indicating there was no significant phototoxicity during 3D two-photon scanning. Box-and-whisker plots show the median, 25th and 75th percentiles, range of nonoutliers and outliers in panels **b**-**g**.

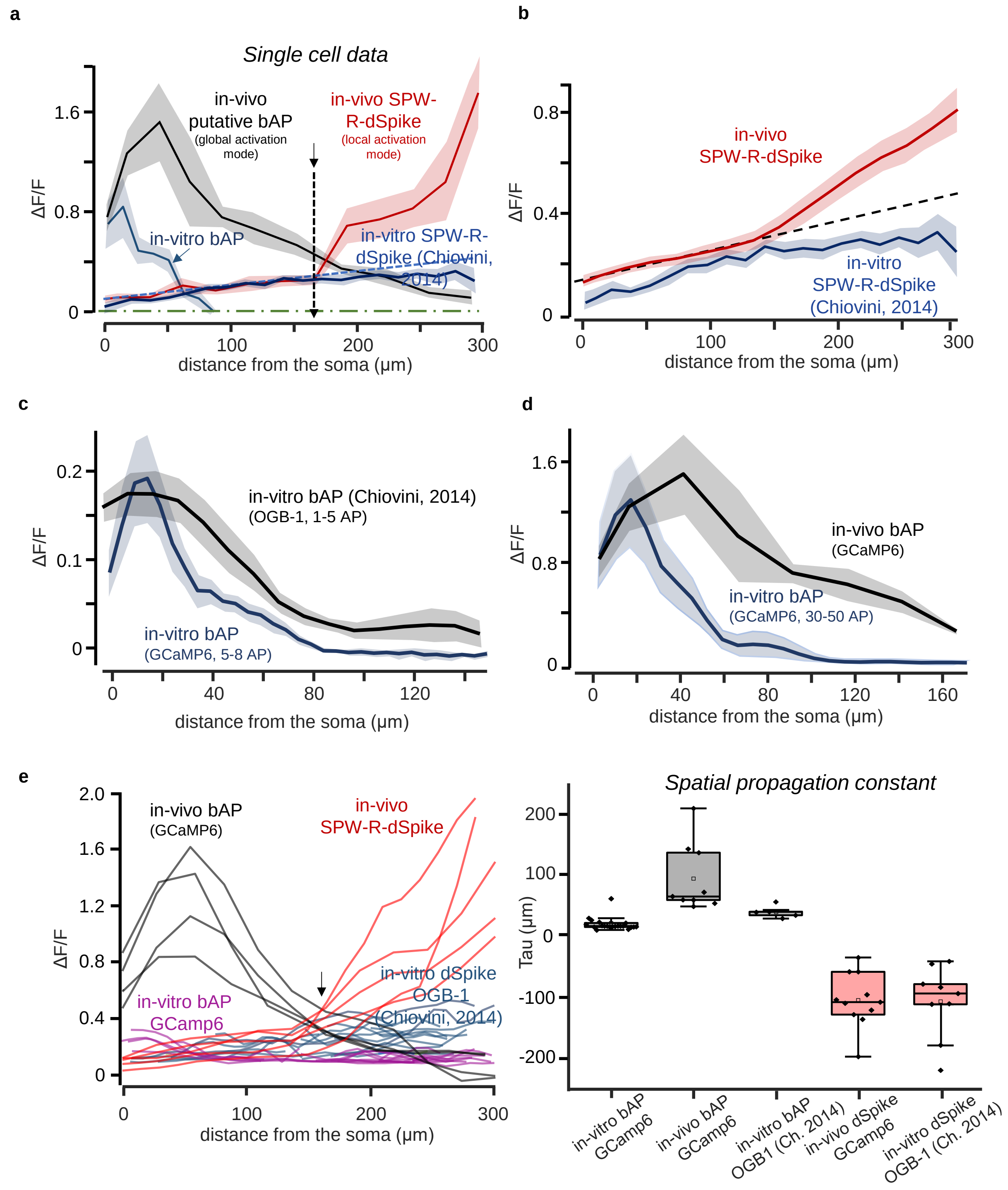

Supplementary Figure 4, Characterization of the global and local activation modes along the somatodendritic axis. a, Same as **Fig. 2c** but spatial distribution of in vitro SPW-R-dSpikes is also shown. Dashed blue line indicates linear fit to the dendritic regions situated between the spatial threshold and the soma (in vitro: n=28/5 traces/cells). **b,** Spatial distribution of the average SPW-R-dSpike-associated dendritic 3D Ca^{2+} responses in vitro (blue) and in vivo (red). Dashed line indicates linear fit to the in vivo proximal dendritic regions (n=11/5 cells/mice). **c,** Spatial distribution of the average bAP-induced dendritic Ca^{2+} responses during in vitro

measurements using OGB-1 Ca^{2+} dye (black) and the GCaMP6f indicator (blue), at low and high numbers of APs, respectively. Note the similar spatial decay constants (in vitro GCaMP6f: n=7/7 events/cells, OGB-1: n=18/5 events/cells). **d,** Spatial distribution of the average bAP-induced dendritic 3D Ca^{2+} responses in vitro and in vivo at high numbers of APs (in vitro GCamp6: n=19/3 events/cells; in vivo Gcamp6: n=4 events). Data are presented as mean± SEM in panels **a-d**. **e,** Left, individual traces during in vivo measurements (bAP: black, n=4 measurement, SPW-R-dSpike: red, n=5 measurement) were completed with individual in vitro responses (in vitro bAP: purple, n=11 measurement, in vitro SPW-R-dSpike: blue, n=9 measurement). Note that dendrites are more active in vivo: SPW-R-dSpikes have much larger amplitude. Moreover, the spatial threshold of the spike (black arrow) emerged only in vivo but not in vitro. Right, box-and-whisker plot indicates the corresponding spatial decay constants. Black diamonds represent individual data points. Note that all bAP-induced responses have positive decay constants, and all SPW-R-dSpikes have negative decay constants, independent of the dyes used. Box-and-whisker plots show the median, 25th and 75th percentiles, range of nonoutliers and outliers, (in vitro bAP GCamp6/in vivo bAP GCam6/in vitro bAP OGB1/in vivo dspike GCamp6/in vitro dspike OGB1 consist of n=22/9/5/11/9 traces).

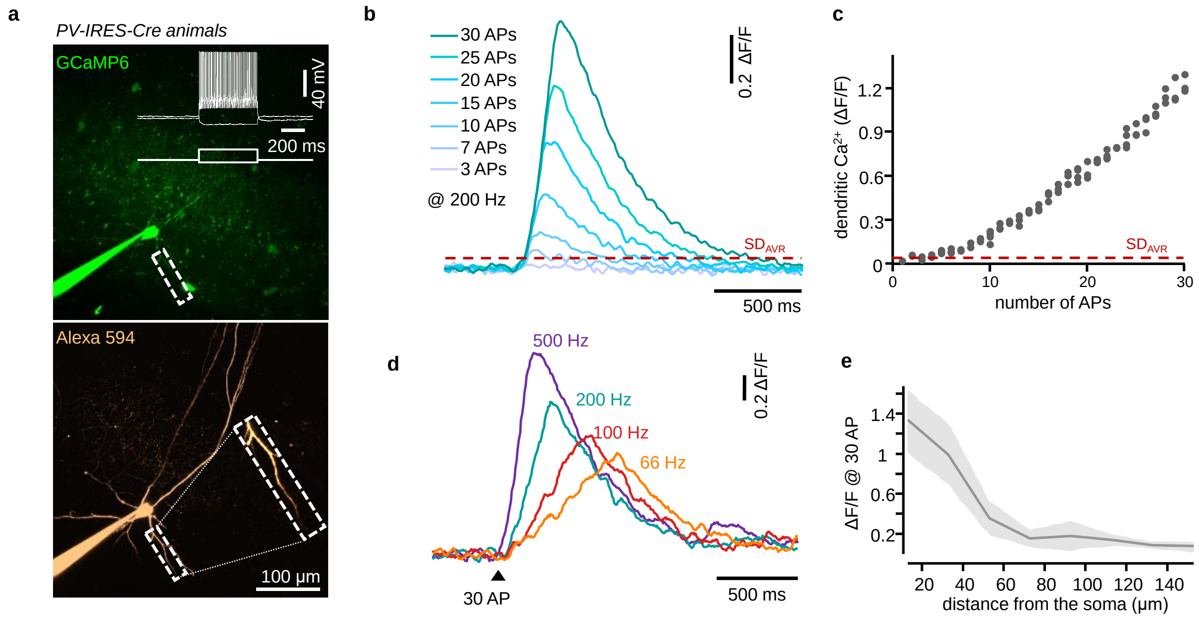

Supplementary Figure 5, Action potential backpropagation into the dendrites of PV+ interneurons. a, Maximum intensity projection of a GCaMP6f-labelled PV+ neuron from 23 plane. The cell was filled with Alexa 594 indicator through the somatic patch electrodes during in vitro recordings. Top, green channel data. Inset, current-clamp recording during somatic current injection steps were used to measure firing rate for validation of fast-spiking PV+ cells. Bottom, red channel data. Lower inset enlarged view shows the recorded stratum oriens dendrite. **b,** Backpropagating AP-induced dendritic Ca^{2+} signals at different AP numbers from the region indicated by the dashed box in panel **a**. APs were induced by somatic current injection at 200 Hz. **c,** Ca^{2+} signals showed a linear increase following an initial sublinear period. The average amplitudes of SPW-R-dSpikes from the high-ripple group and from the group of doublets were 44% (ΔF/F) and 130% (ΔF/F), respectively (see **Fig. 4**), being in the range of data in panel **c**. Therefore, dendritic responses remained linear in PV+ cells even in the high amplitude range characteristic for SPW-R-dSpikes. **b-c,** Dashed red line indicates the average SD value (SD_{AVR}) of dendritic measurements (n=10). **d,** Frequency dependency of the dendritic Ca^{2+} transients evoked by 30 APs. The higher response at 500 Hz vs. 200 Hz also indicated that the GCaMP6f sensor was not saturated in these measurements. **e,** Distance-dependent mean amplitude of the Ca^{2+} response along the dendrite at 30 APs (mean ± SEM, n=5 measurements). Backpropagating AP-induced dendritic Ca^{2+} responses decreased rapidly as a function of distance (see also **Supplementary Figure 3** for a detailed comparison).

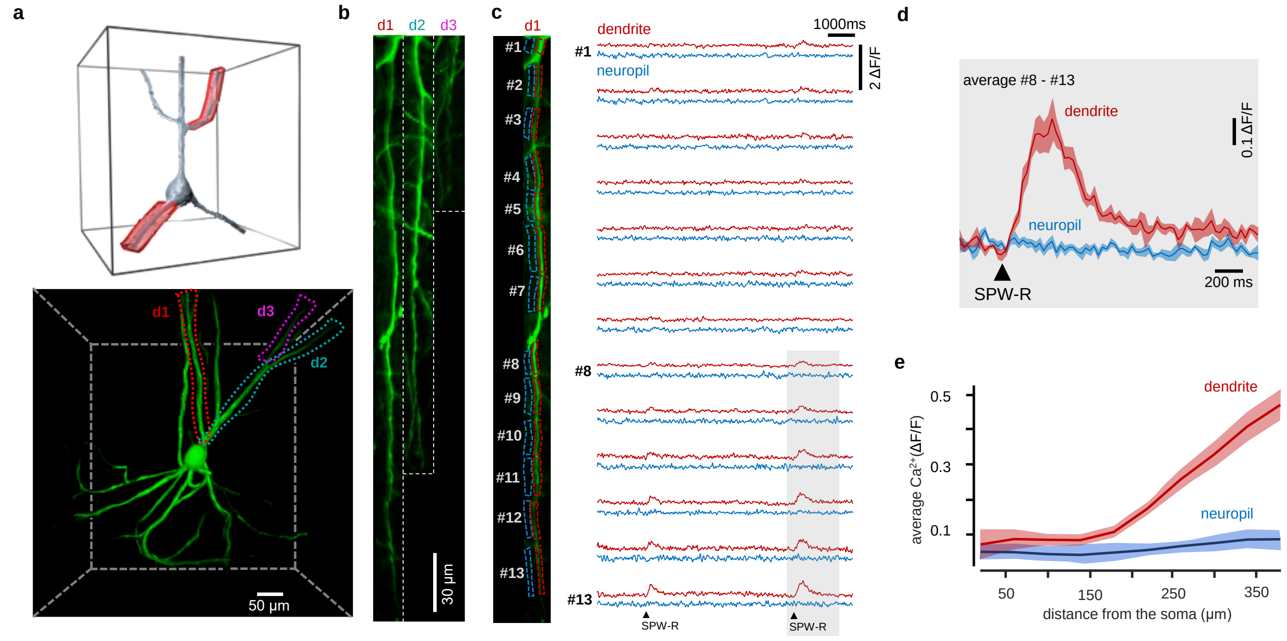

Supplementary Figure 6, Neuropil signalization and background did not contribute to the distance-dependent increase in SPW-R-dSpike amplitude recorded in 3D. a, Top, schematic of the measurement. Bottom, 3D visualization of a GCaMP6f-labelled PV+ cell from a PV-IRES-Cre animal from 36 plane. d1, d2 and d3 indicate three dendritic segments measured with 3D ribbon scanning. **b,** Fluorescence data were projected into 2D and shown as a function of transverse and perpendicular distances along the surface of the three ribbons; the three regions were aligned next to one other. The projection of this representative dendrites consists of 603 frames and measured over 10 seconds. **c**, Left, dendritic ROIs selected on the d1 dendrite (red dashed boxes) with the surrounding neuropil (blue dashed boxes). ROIs were defined to avoid segments with the neuropil crossing the d1 dendrite. Right, individual Ca^{2+} transients calculated from the numbered red and blue regions shown on the left. Black arrowheads indicate SPW-R events. Note the dSpikes in distal dendritic segments during SPW-Rs. **d,** Ca^{2+} transients (red, mean ± SEM) were averaged from dendritic regions #8–#13 from the grey box in **c**. Simultaneously-recorded neuropil signals (blue) averaged from the adjacent background areas (mean ± SEM). Our data indicate that neuropil signals did not, on average, contribute to SPW-R-dSpikes. **e,** Mean amplitude of the Ca^{2+} response along dendrite d1 and from the neighbouring neuropil regions during an SPW-R event as a function of distance from the soma.

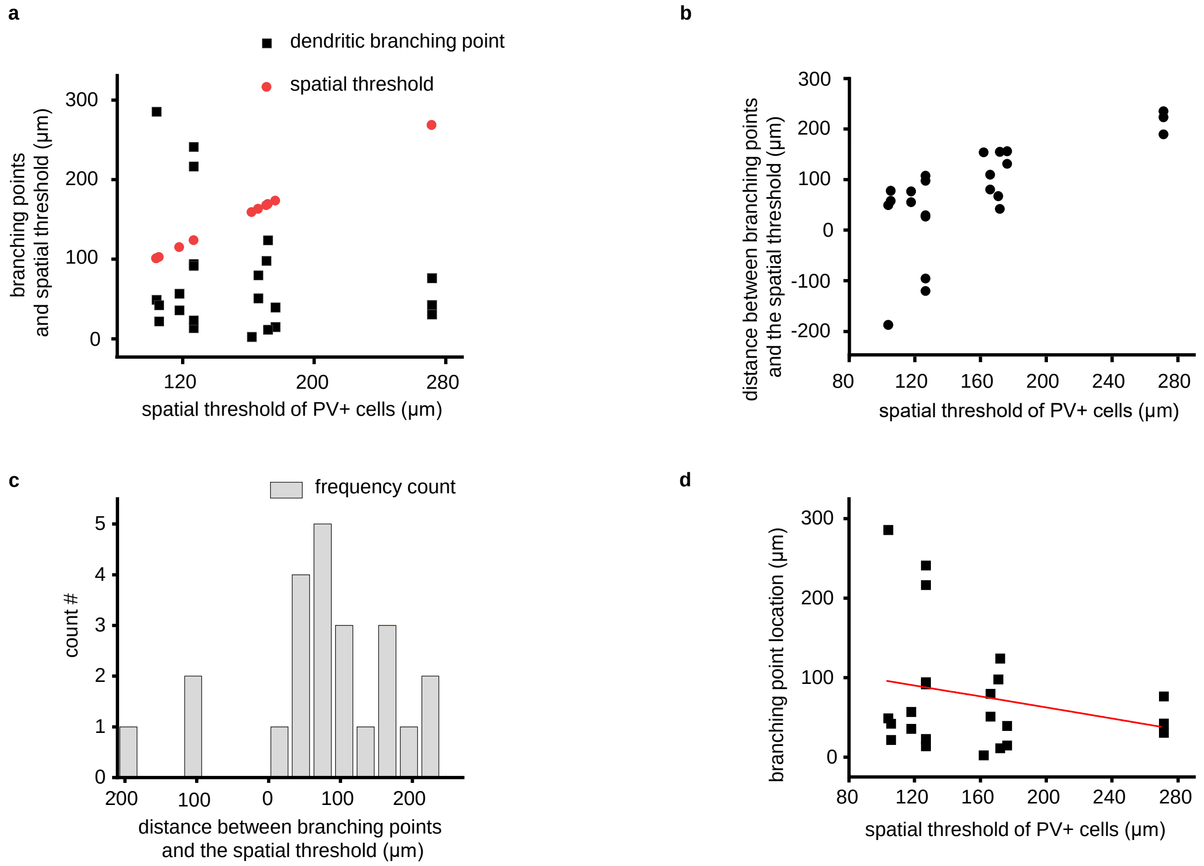

Supplementary Figure 7, Location of dendritic branching points and the spatial threshold of the in vivo SPW-R-dSpike in individual PV+ neurons. a, Location of dendritic branching points (black) and the spatial threshold (red) as a function of the spatial threshold. **b,** Distance of the dendritic branching point and the spatial threshold of the SPW-R-dSpike as a function of the spatial threshold. **c,** Binned histogram of panel **b**. **d,** Correlation between branching point location and the spatial threshold of the SPW-R-dSpike. Pearson's r value: – 0.24.

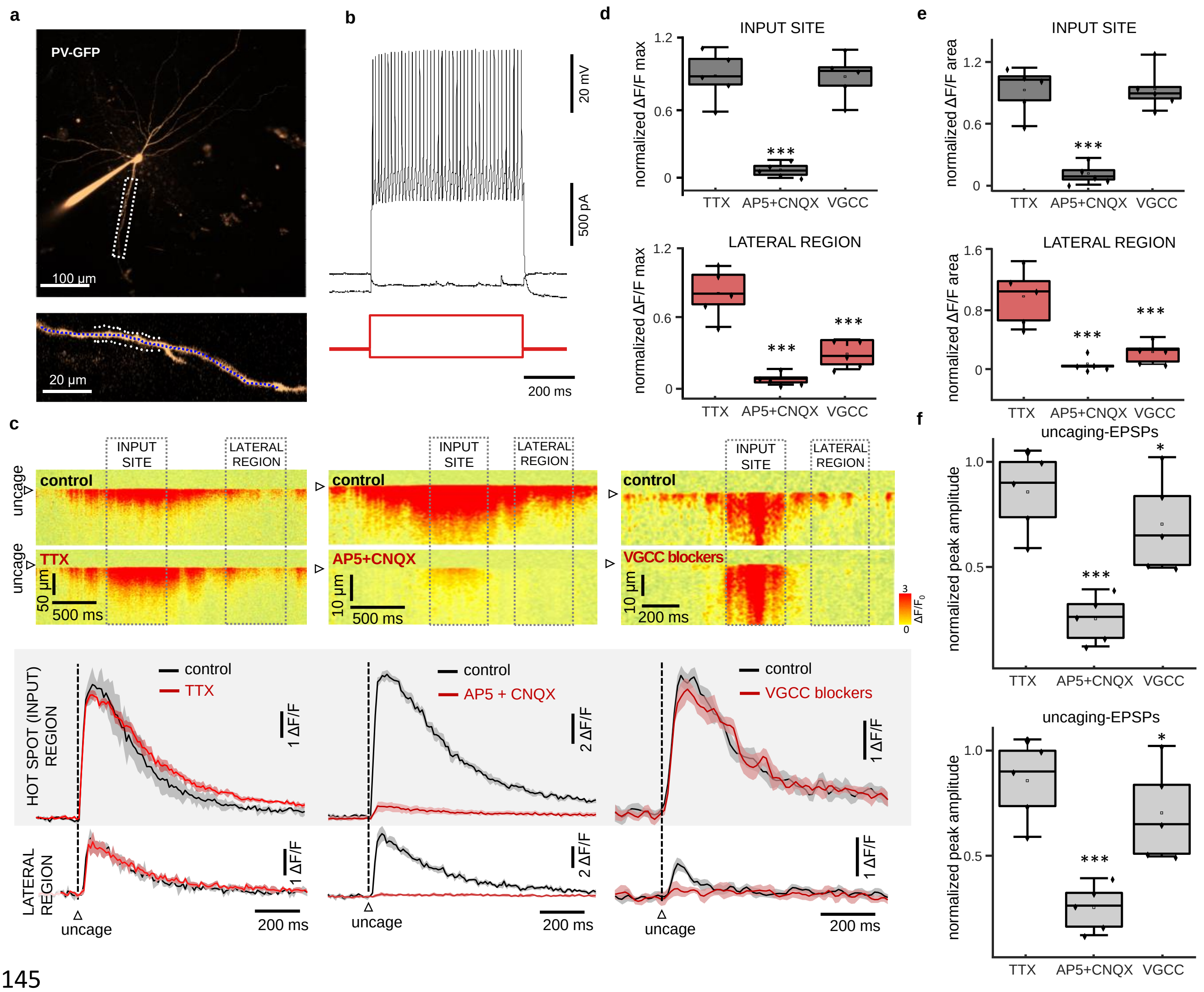

Supplementary Figure 8, dSpikes are dominantly mediated by voltage-gated Ca^{2+} channels. **a,** Top, maximal-intensity projection (from 48 plane) of a CA1 PV+ interneuron was filled with Fluo-4 Ca^{2+} indicator through a patch pipette used for in vitro whole-cell recording. White dashed box indicates a segment of a stratum oriens dendrite selected for simultaneous 3D imaging and photostimulation. Bottom, enlarged view of the selected dendritic segment with white dots indicating input sites where DNI glutamate was uncaged. Laser intensity and photoactivation time was set at each input site to induce somatic EPSPs with an amplitude of unitary responses (**Methods**). Then this spatially-clustered pattern of inputs was activated to mimic SPW-R-dSpikes. Blue dashed curve indicates location of fast 3D scanning. **b,** Ramp test of the PV+ cell shown in **a**. Top, somatic membrane potential responses. Bottom, corresponding somatic current injection. **c,** Top, uncaging-evoked dendritic Ca^{2+} responses recorded along the blue dashed line in **a** under control conditions and in the presence of different ion-channel blockers (the sodium-channel blocker tetrodotoxin: TTX; the AMPA- and NMDA-channel blockers CNQX and AP5; and the cocktail of VGCC blockers: mibefradil, nimodipine and ω-conotoxin). DNI-glutamate was uncaged along the white dots in **a**. Triangles indicate uncaging time. Bottom, Ca^{2+} transients derived from the central input and lateral

dendritic regions shown in the top panels. **d-e,** Corresponding peak amplitudes and areas (n=5 mice per group, data points showing the individual activity). **f,** The same as panel **d-e**, but for the simultaneously-recorded somatic EPSPs. In summary, while AP5 and CNQX effectively blocked the initiation and the central component of the dSpikes, the cocktail of VGCC blockers eliminated the centrifugally and centripetally propagating component of the dSpike in the lateral dendritic regions and left the central component of the dSpike unaffected (Student's two-way paired t-test; * $p<0.05$, ** $p<0.01$, *** $p<0.001$). Box-and-whisker plots (**d-f**) show the median, 25th and 75th percentiles, range of nonoutliers and outliers (**d-f**).

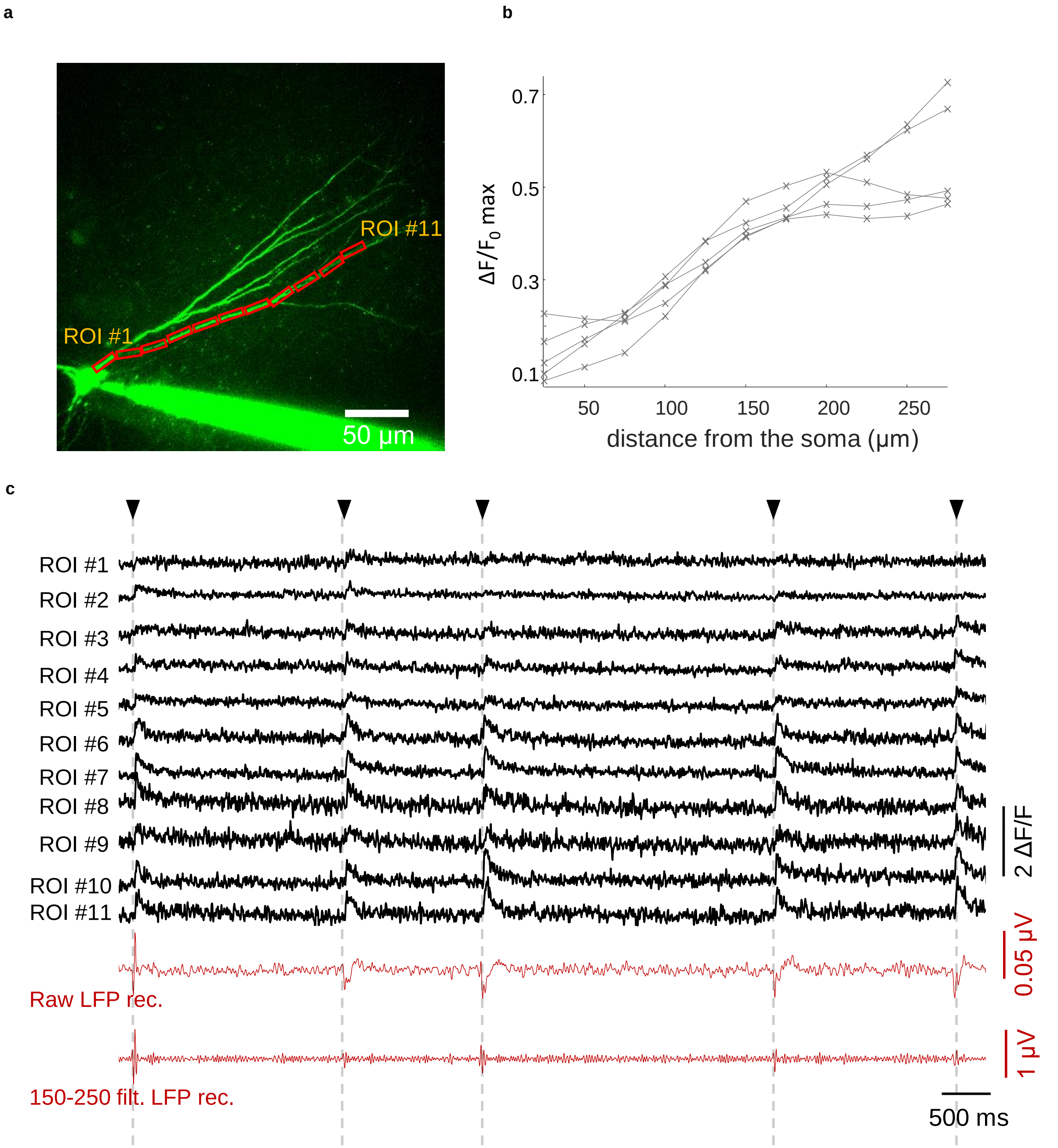

Supplementary Figure 9, Spatial distribution of SPW-R-dSpike-associated dendritic Ca^{2+} responses recorded in 3D in vitro. a, Maximal-intensity z-projection from 26 plane of an Oregon green BAPTA-1-labelled CA1 PV^{+} interneuron in a PV-GFP mouse. The neuron was loaded through the patch electrode. Red boxes indicate 11 ROIs (regions of interest) along a stratum oriens dendrite. **b**, Spatial distribution of the amplitude of the individual SPW-associated dendritic Ca^{2+} responses. The amplitude of Ca^{2+} responses increased as a function of distance from the soma. **c**, Individual Ca^{2+} responses spatially averaged for the numbered dendritic regions indicated in **a** show a distance-dependent increase during SPW-Rs. Arrowheads and dashed lines indicate SPW-R events.

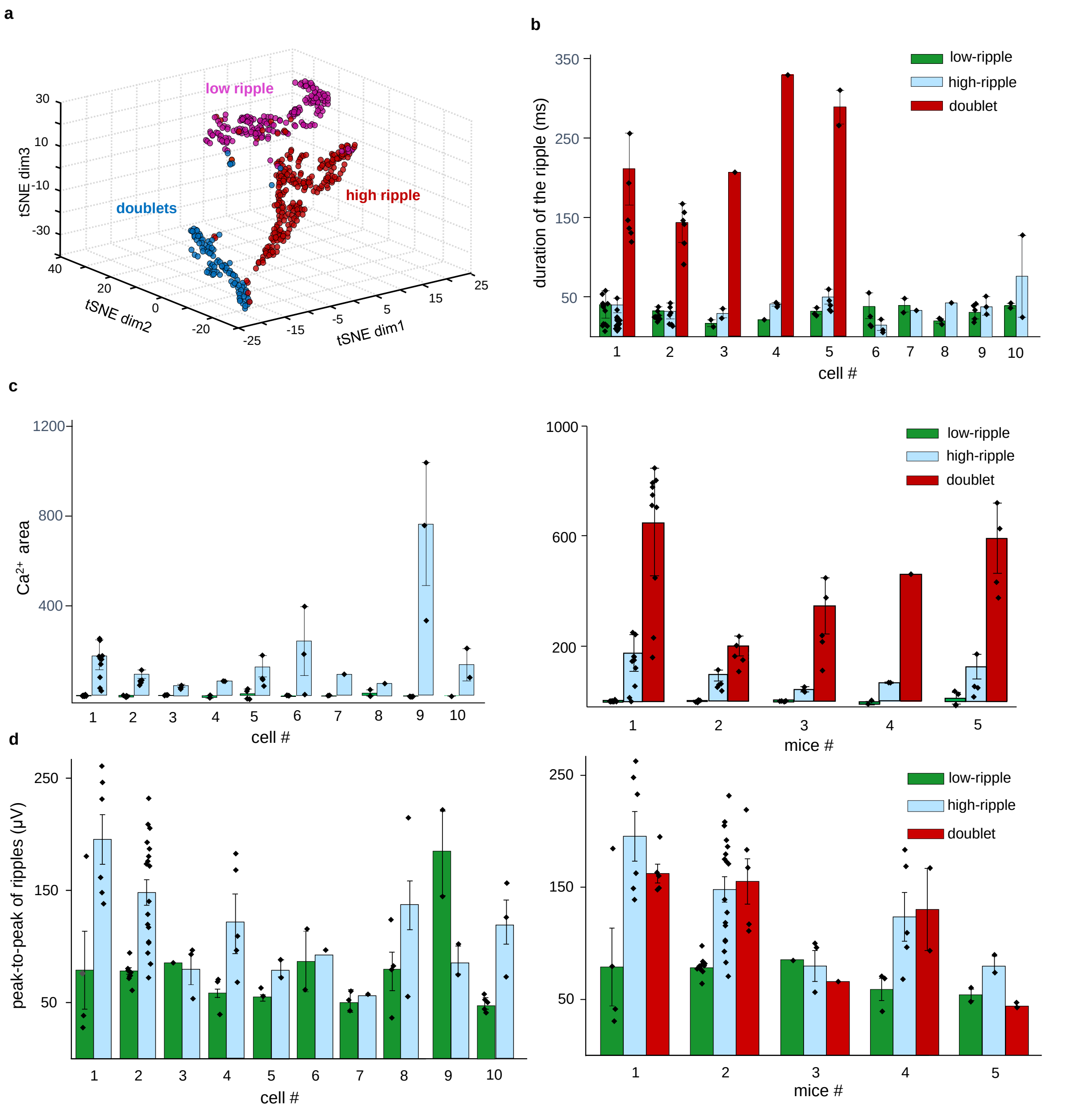

Supplementary Figure 10, Statistical differences between the three different groups (low-ripple, high-ripple, and doublets) across cells and animals. a, The t-distributed stochastic neighbor embedding cluster analysis (tSNE) was used on the extended database of the SPW-R-associated events (n=548/5/5 dendritic segments/cells/mice, Z-score transformation was used on original values). This approach constructs a set of embedded points in a low-dimensional space whose relative similarities mimicked those of the original points. tSNE models the original points as originating from a Gaussian distribution, while a Student's distribution is used to model the embedded points. The Kullback-Leibler divergence between these two distributions is minimized by moving the embedded points. **b**, Duration of ripple oscillation during low-ripples (green), high-ripples (blue), and doublets (red) in individual neurons (n=10/5 cells/mice). Duration was not significantly different between the low- and high-ripple groups at the level of individual neurons, but lasted significantly longer according to the population data (**Fig. 3a**). **c**, Left, dendritic Ca^{2+} responses (areas) of individual neurons

during high-ripples were higher than in the low-ripple events (n=10 cells). Right, the same but with doublets for n=5 mice. These data reflect the population averages (**Fig. 4f-h**) and spectral histograms (**Fig. 3a**). **d**, Left, peak-to-peak amplitudes of ripples during high-ripple events were significantly higher than during low-ripple events in n=5 cells, mirroring the population averages and spectral histograms of the population data (**Fig. 3a**, **Supplementary Figure 4g**). Right, the same as panel **a** but with doublets for n=5 mice. Data are presented as mean ±SEM in panels **b**-**d**.

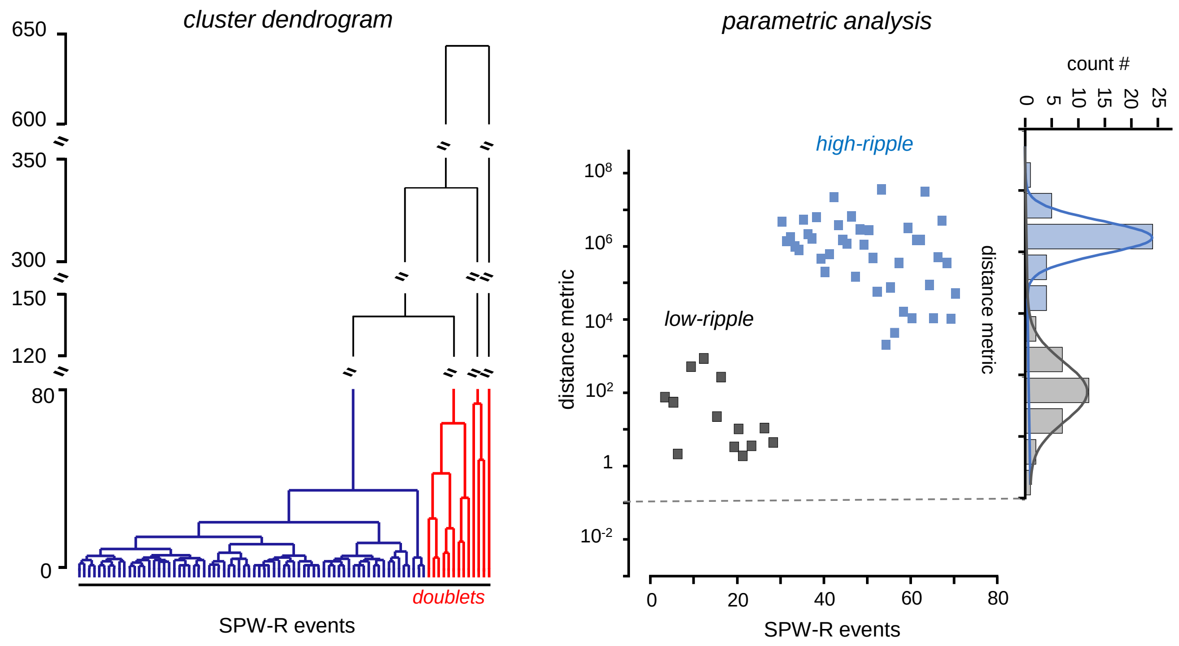

Supplementary Figure 11, Separation of SPW-Rs and the simultaneously-recorded dendritic responses with combined cluster analysis and parametric analysis. Left, cluster analysis of the simultaneously recorded SPW-R-associated LFP and 3D Ca^{2+} signals separated a group in which two SPW-R events followed each other within a short time window (SPW-R doublets, red, n=11/5 cells/mice). Right, a successive parametric analysis in the next step separated the rest of the responses (dark blue) into two groups: low-ripple (grey) and high-ripple (light blue) events. Inset, y-axis histogram: projection of the distance metric data. Multiple Gaussian fit (blue and grey curves) validated that the high- and low-ripple groups are two independent populations. The Peak Analyser module (Origin Pro, OriginLab) identified two Gaussian distributions ($\chi 2$=2.12, adj. R-Square=0.95, SS=12.27, coefficient of determination = 97.4%; see Supplemental Methods for details) corresponding to the high ripple (blue) and the low ripple (black) groups.

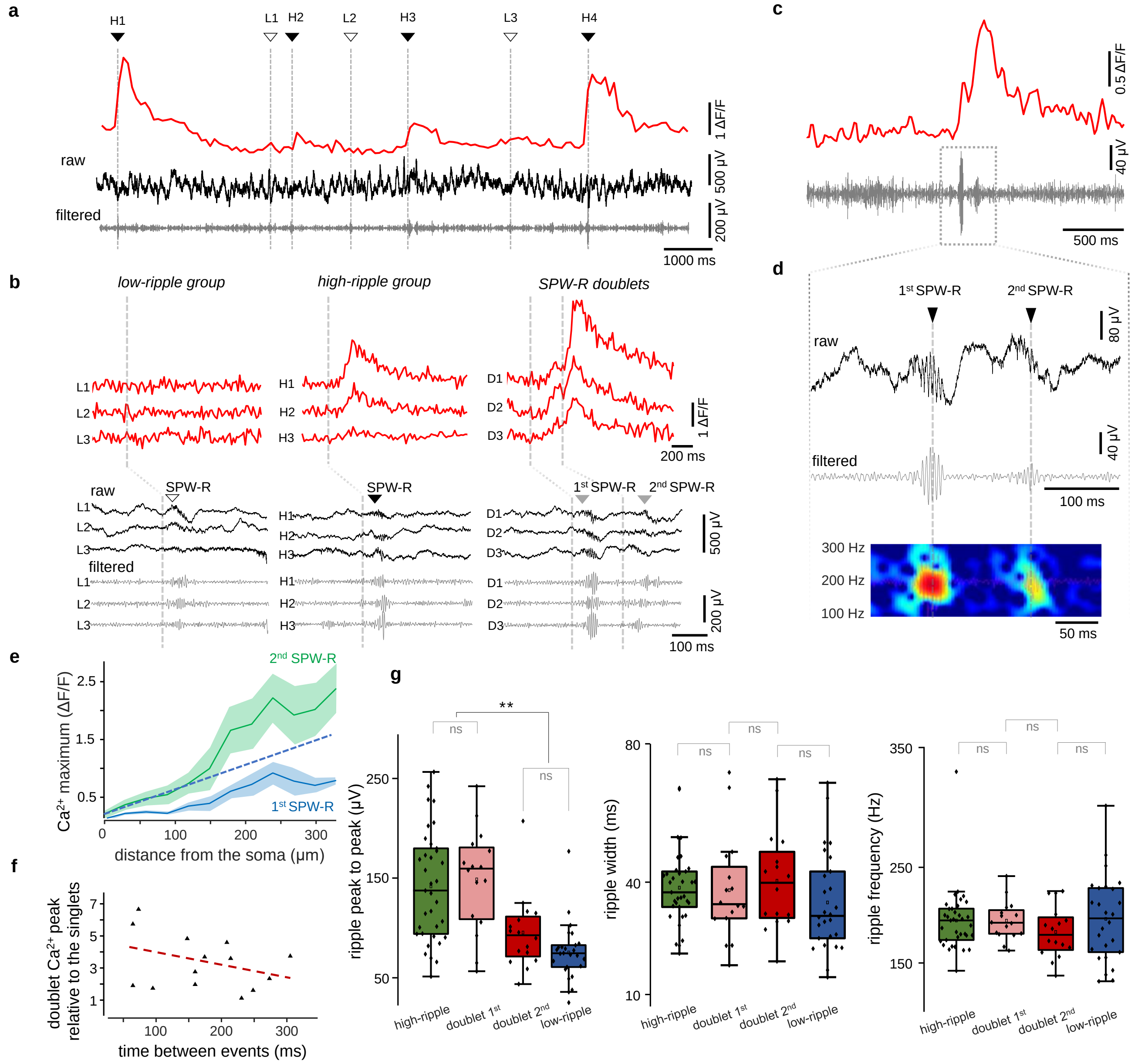

Supplementary Figure 12, SPW-R-associated LFP signals and dendritic Ca^{2+} responses form three groups during in vivo recordings. a, Spatially-averaged Ca^{2+} response from a distal dendritic segment (red) and simultaneously-recorded local LFP (raw signals: black, filtered: grey) with alternating events from the high- (filled triangles) and low- (empty triangles) ripple groups from a PV+ interneuron. **b**, Exemplified individual events from the low- and high-ripple groups and the group of SPW-R-doublets from the same PV+ cell. Raw (black traces) and filtered (grey traces) LFP signals are shown with the simultaneously-recorded Ca^{2+} responses. **c**, Exemplified SPW-R doublet event, where the dendritic Ca^{2+} response (red) and simultaneously-recorded local LFP signal (grey) are shown. **d**, Top, magnified view of the grey dashed box in **c** with the raw LFP data (black) showing the first and second events of the SPW-R doublet (black triangles). Bottom, spectrogram of the SPW-R doublet. **e**, Average amplitude of the dendritic Ca^{2+} response during the first (blue) and the second (green) event of the SPW-

R doublets as a function of distance from the soma. Dashed blue line indicates linear fit to the proximal dendritic regions. Note the sharp increase in the response amplitude above the spatial threshold. **f**, Normalized amplitude of the SPW-R doublet-associated dendritic Ca^{2+} responses as a function of the time delay between the first and second events. Amplitude of SPW-R doublets was normalized to the average amplitude of singlets in each cell (n=5/5 cells/mice). **g**, Box-and-whisker plots show the median, 25th and 75th percentiles, range of nonoutliers and outliers of the ripple peak-to-peak amplitude, ripple width, and ripple frequency for the SPW-R-associated events from the high- and low-ripple groups, and for the first and second peak of doublets (n=77/5/5 traces/cells/mice, Student's one-way paired t-test; **p<0.01).

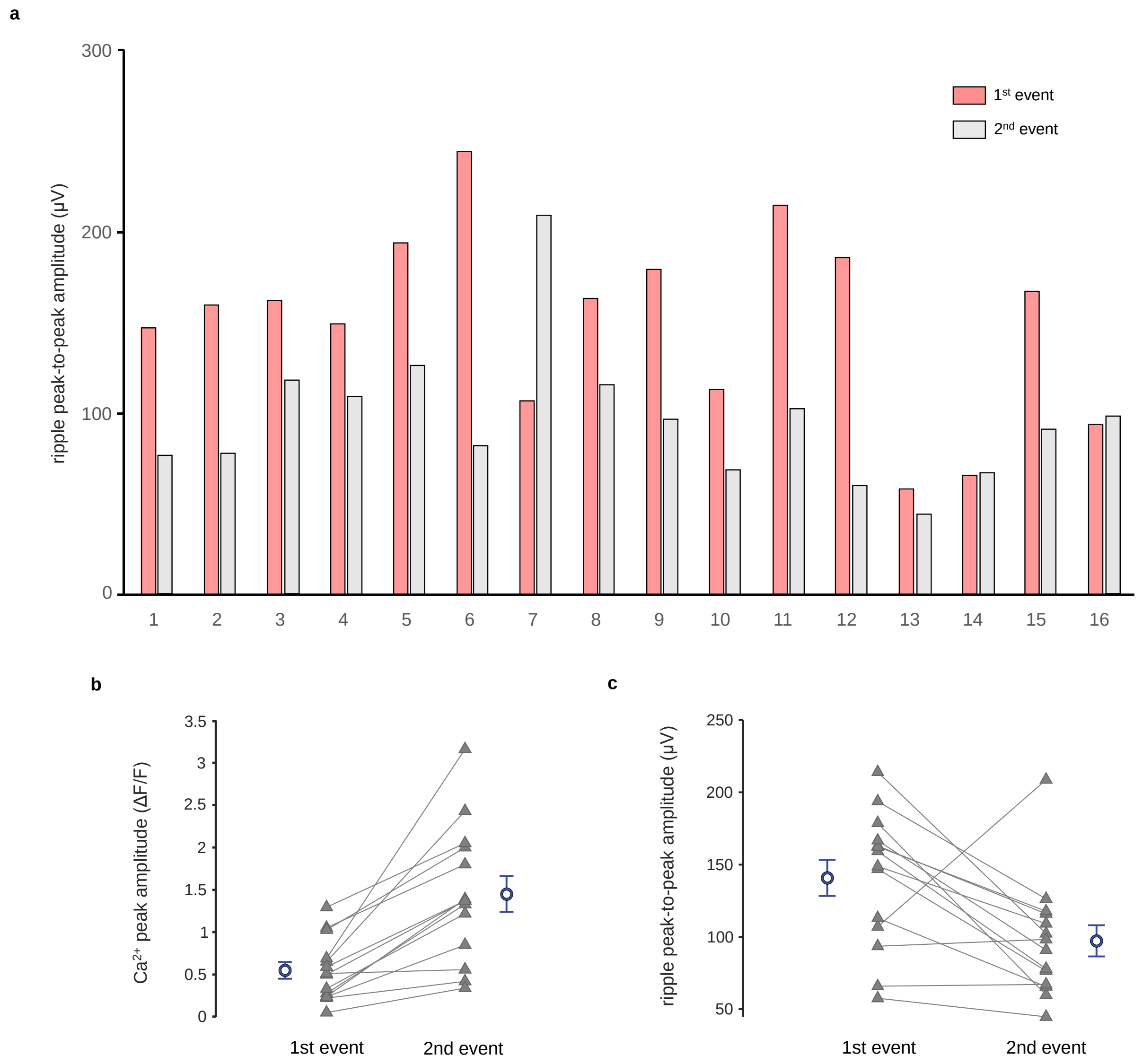

Supplementary Figure 13, Characterization of the 1st and 2nd events of SPW-R-doublets. a, Peak-to-peak amplitude of the 1st and 2nd ripple events of SPW-R-doublets. **b,** Corresponding peak Ca^{2+} responses of the doublets. Note that the 2nd components were equal to or higher than the 1st for each doublet. **c,** Ripple peak-to-peak amplitude for the 1st and 2nd events of the doublets. There was only one doublet where the second event was larger than the first. Circles and error bars indicate mean ± SEM.

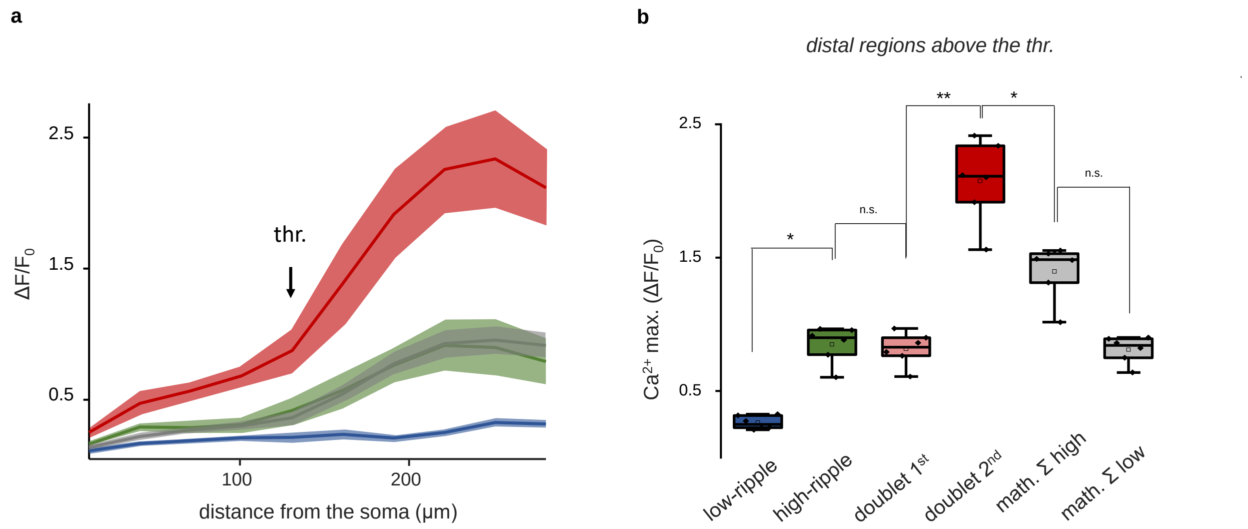

Supplementary Figure 14, Dendritic supralinearity during SPW-R-doublet-dSpike can be detected at cellular level. a, The same PV+ neuron as in **Fig. 4b** but with the mean±SEM values of the individual responses (n=18/12/6 high ripples/low ripples/doublets). Black arrow indicates spatial threshold. Gray curve is the average (mean±SEM) mathematical sum of the high- and low-ripple events. Note the robust supralinear summation above the spatial threshold. **b**, Similar to **Fig. 4e**, box-and-whisker plots calculated from data shown in panel **a** using 25-µm bins above the spatial threshold of the spike (Student's one-way paired t-test; *** $p<0.001$, n=6 dendritic segment in each group). Box-and-whisker plots show the median, 25th and 75th percentiles, range of nonoutliers and outliers.

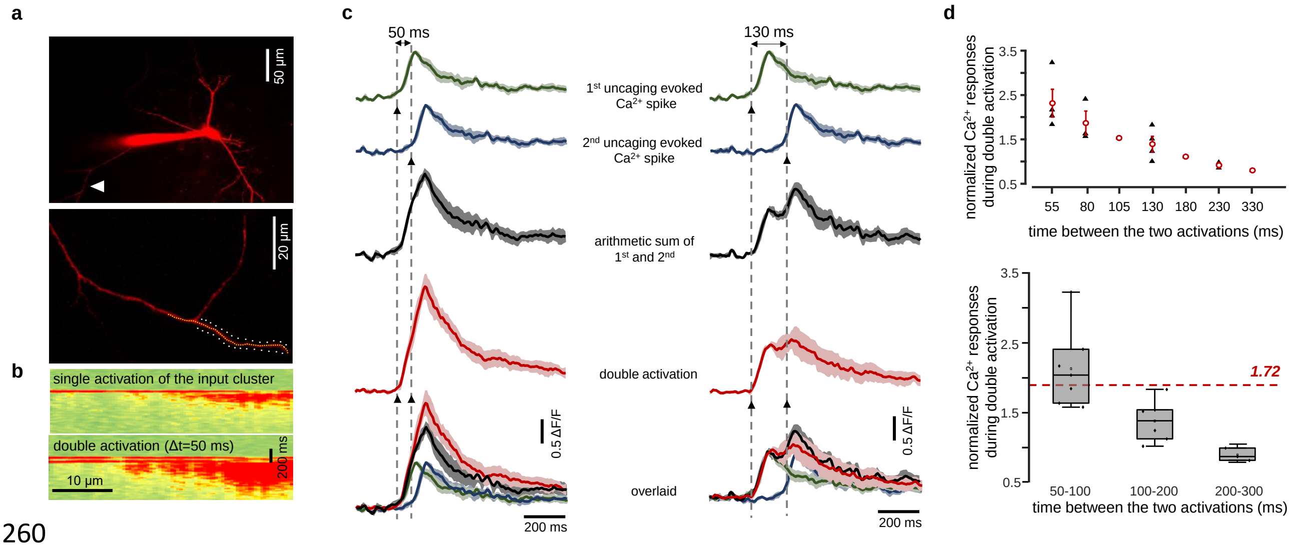

Supplementary Figure 15, Doublets of SPW-R-dSpikes reproduced with clustered input patterns show timing-dependent supralinear summation. a, Top, maximum-intensity z-projection of a PV+ interneuron from 66 plane, filled with the Fluo-4 Ca^{2+} indicator and Alexa 594 fluorescent dye. Arrowhead indicates the stratum oriens dendritic segment measured in vitro. Bottom, enlarged view of the imaged dendritic segment. White dots and yellow dashed line indicate the location of DNI glutamate uncaging and 3D Ca^{2+} imaging, respectively. **b,** Top, dendritic responses along the yellow dashed line shown in panel **a (**90 binned dendritic region). Number of inputs activated with DNI-glutamate uncaging was set to reproduce the amplitude of SPW-R-dSpikes from the high-ripple group (see **Fig. 3**). Bottom, the same input cluster was activated two times with a 50 ms delay (double activation) to mimic SPW-R-dSpikes (90 binned dendritic region). Note the much larger dendritic responses during double activations. **c,** Supralinear Ca^{2+} summation during double activation. Green and blue traces are average dendritic responses (mean ± SEM) during activation of the input cluster only once (1st and 2nd uncaging evoked Ca^{2+} spikes, respectively). Blue traces were shifted with 50 ms (left) and 130 ms delay (right). Grey traces are the mathematical sum of the blue and green traces (mean ± SEM); red transients are the average (mean ± SEM) responses during the double activation when the input cluster was activated two times with a 50 ms (left) and 130 ms delay (right). Bottom, overlaid transients show supralinear dendritic integration during the double activation. Dashed lines indicate timing of the first and second activations (n=16 measurements, n=4 mice). **d,** Top, amplitude of dendritic Ca^{2+} responses during dSpikes as a function of the time between the two activations (black: individual responses, red: mean ± SEM). Responses were normalised to the amplitude of dSpikes induced by single activation. Bottom, pooled dendritic responses at different timing intervals. The same intervals were used as in **Fig. 4j**. The red dashed line indicates the amplitude of the mathematical sum (which is the threshold of supralinear summation). Box-and-whisker plots show the median, 25th and 75th percentiles, range of nonoutliers and outliers, (n=16 measurements, n=4 mice).

Supplementary Methods

SPW-R parameter product-based distance metric to separate SPW-R-associated responses

As a first step towards separating SPW-Rs and the simultaneously-recorded dendritic responses into multiple functional subpopulations, we characterized these events with eleven parameters: the amplitude and area of the spatially averaged 3D dendritic Ca^{2+}responses; the amplitude and half-width of the depolarizing envelop of the SPW-R events; the maximal and minimal amplitude, the peak-to-peak amplitude, the duration, the power, and the area of absolute value the ripple oscillations; and the number of cycles within the ripple oscillations. The second step was to perform cluster analysis in OriginPro (OriginLab) which separated the group of SPW-R doublets from solitary SPW-R events (**Supplementary Figure 11a**). However, the rest of the events remained as one homogeneous group (solitary SPW-R events) and neither cluster nor principal component analysis was able to split them into subgroups. The third step was to rank the solitary SPW-R events according to their dendritic response amplitude and divide them into two subgroups (small and large events). Then we selected the first six parameters from the eleven listed above: these showed the highest change between the small and the large event groups relative to the standard deviation of the parameter. In the next step, all information related to the SPW-R-associated data was ignored and we analysed only the selected six parameters to avoid circularity. We multiplied the six parameters and used the logarithmic function to generate a distance metric with which the existence of subgroups was measured (**Supplementary Figure 11a**). Gaussian peak fitting by Quick Peaks (OriginPro, OriginLab) to the binned distance data identified two distinct subpopulations within the group of solitary SPW-R events: the low- and high-ripple groups (coefficient of determination: 0.97, reduced χ^2: 2.11, **Supplementary Figure 11a**) as shown in the histogram of the distance parameters (**Supplementary Figure 11a, inset**). If not otherwise indicated, data are shown as means ± SEM and we used the Student's t-test (* $p<0.05$, ** $p<0.01$, or *** $p<0.001$).

Supplementary References

1. Muldoon, S. F. *et al.* GABAergic inhibition shapes interictal dynamics in awake epileptic mice. *Brain J. Neurol.* **138**, 2875–2890 (2015).
2. Villette, V., Levesque, M., Miled, A., Gosselin, B. & Topolnik, L. Simple platform for chronic imaging of hippocampal activity during spontaneous behaviour in an awake mouse. *Sci. Rep.* **7**, 43388 (2017).
3. Farrell, J. S. *et al.* In vivo assessment of mechanisms underlying the neurovascular basis of postictal amnesia. *Sci. Rep.* **10**, 14992 (2020).
4. Geiller, T. *et al.* Large-Scale 3D Two-Photon Imaging of Molecularly Identified CA1 Interneuron Dynamics in Behaving Mice. *Neuron* **108**, 968-983.e9 (2020).
5. Grosmark, A. D., Sparks, F. T., Davis, M. J. & Losonczy, A. *Offline Memory Reactivation Promotes the Consolidation Of Spatially Unbiased Long-Term Cognitive Maps*. http://biorxiv.org/lookup/doi/10.1101/2020.08.20.259879 (2020) doi:10.1101/2020.08.20.259879.
6. Sparks, F. T. *et al.* Hippocampal adult-born granule cells drive network activity in a mouse model of chronic temporal lobe epilepsy. *Nat. Commun.* **11**, 6138 (2020).
