# Supplementary material for: Sharp-wave ripple doublets induce complex dendritic spikes in parvalbumin interneurons in vivo
Source: Nat Commun. 2022 Nov 7;13:6715. doi: 10.1038/s41467-022-34520-1 (PMC9640570; doi:10.1038/s41467-022-34520-1)
Supplement: Supplementary file 2 — Description of Additional Supplementary Files [file 41467_2022_34520_MOESM2_ESM.pdf]

File name: Supplementary Movie 1

Description: related to Fig. 1g-h, Simultaneous imaging of multiple dendritic segments with 3D ribbon scanning with motion artefact compensation. The video demonstrates the use of 3D ribbon scanning on a model neuron. We fitted long ribbons in 3D along the dendrites of the cells (red), then scanned along the surfaces of the ribbons. In the next step, the fluorescent data were projected into 2D and shown as a function of transverse and perpendicular distances along the surface of the ribbons. Finally, motion artifacts were eliminated by shifting back each frame of all regions by the local projection of the net displacement vector of the brain.

File name: Supplementary Movie 2

Description: related to Fig. 1h and 2a-b. 3D ribbon scanning of CA1 PV+ cell dendrites revealed dendritic spikes during SPW-Rs in awake mice. We simultaneously measured 10 dendrites of a GCaMP6f-labeled CA1 PV+ cell shown in Fig. 1h using 3D ribbon scanning in parallel with LFP recording. Note, when SPW-R events were detected, dendritic spikes occurred only in the distal part of the first dendrite.
