# Supplementary material for: Sharp-wave ripple doublets induce complex dendritic spikes in parvalbumin interneurons in vivo
Source: Nat Commun. 2022 Nov 7;13:6715. doi: 10.1038/s41467-022-34520-1 (PMC9640570; doi:10.1038/s41467-022-34520-1)
Supplement: Supplementary file 5 — Reporting Summary [file 41467_2022_34520_MOESM5_ESM.pdf]

nature portfolio

Corresponding author(s): Dr.Balázs J. Rózsa

Last updated by author(s): 2022.10.27.

Reporting Summary

Nature Portfolio wishes to improve the reproducibility of the work that we publish. This form provides structure for consistency and transparency in reporting. For further information on Nature Portfolio policies, see our Editorial Policies and the Editorial Policy Checklist.

Statistics

For all statistical analyses, confirm that the following items are present in the figure legend, table legend, main text, or Methods section.

n/a	Confirmed	
☐	☒	The exact sample size (n) for each experimental group/condition, given as a discrete number and unit of measurement
☐	☒	A statement on whether measurements were taken from distinct samples or whether the same sample was measured repeatedly
☐	☒	The statistical test(s) used AND whether they are one- or two-sided<br>*Only common tests should be described solely by name; describe more complex techniques in the Methods section.*
☒	☐	A description of all covariates tested
☒	☐	A description of any assumptions or corrections, such as tests of normality and adjustment for multiple comparisons
☐	☒	A full description of the statistical parameters including central tendency (e.g. means) or other basic estimates (e.g. regression coefficient) AND variation (e.g. standard deviation) or associated estimates of uncertainty (e.g. confidence intervals)
☐	☒	For null hypothesis testing, the test statistic (e.g. F, t, r) with confidence intervals, effect sizes, degrees of freedom and P value noted<br>*Give P values as exact values whenever suitable.*
☒	☐	For Bayesian analysis, information on the choice of priors and Markov chain Monte Carlo settings
☒	☐	For hierarchical and complex designs, identification of the appropriate level for tests and full reporting of outcomes
☒	☐	Estimates of effect sizes (e.g. Cohen's d, Pearson's r), indicating how they were calculated

Our web collection on statistics for biologists contains articles on many of the points above.

Software and code

Policy information about availability of computer code

Data collection	All two-photon experiment were performed with a 3D-AO laserscaning microscope (ATLAS, Femtonics) and Matlab -based software (MES 5 version 2043-3143, Femtonics), Local (LFP) signal were recorded using Intan RHD 2132 interface board (Intan Technologies, version 1.41) with an amplifier bandwidth of 0.1 Hz to 7.5 kHz and sampled at 20 kHz. Population activity were measured with MESc software (Femtonics, MESc version 3.4).
Data analysis	Calcium imaging data and electrophysiological recordings were analysed using Matlab-based software (MES 5 version 2043-MES 6 version 11391, Femtonics), OriginPro (Originlab) and Excel (Microsoft). ImageJ open-source software (ImageJ 1.47 version) with some custom-written macros to generate projections from the data recorded in 3D.

For manuscripts utilizing custom algorithms or software that are central to the research but not yet described in published literature, software must be made available to editors and reviewers. We strongly encourage code deposition in a community repository (e.g. GitHub). See the Nature Portfolio guidelines for submitting code & software for further information.

Data

Policy information about availability of data

All manuscripts must include a data availability statement. This statement should provide the following information, where applicable:

- Accession codes, unique identifiers, or web links for publicly available datasets
- A description of any restrictions on data availability
- For clinical datasets or third party data, please ensure that the statement adheres to our policy

Because a source data file supporting the findings of this study is provided with this paper and its supplementary information files, therefore the additional binary data files are available from the corresponding author upon request.

12

Field-specific reporting

Please select the one below that is the best fit for your research. If you are not sure, read the appropriate sections before making your selection.

☒ Life sciences ☐ Behavioural & social sciences ☐ Ecological, evolutionary & environmental sciences

For a reference copy of the document with all sections, see nature.com/documents/nr-reporting-summary-flat.pdf

Life sciences study design

All studies must disclose on these points even when the disclosure is negative.

Sample size	No samlpe size calculation was performed. Sample size were chosen based on similar published studies using comparable approaches (J. Cichon et al., 2015; Francavilla et al.,2019; Geiller et al.,2020).
Data exclusions	Data were not excluded from analysis.
Replication	We performed recordings from multiple animals to confirm reproductibility.Replication were successful.i) In the first set of experiments (Fig. 1a-f), we imaged 261 PV+ interneurons in n=4 mice. The percentage of PV+ neurons that were active during SPW-R activity was calculated from n=4 mice (15.1%, i.e. n=13 cells were active during SPW-R events from the n=86 active neurons).<br>ii) In the second set of experiments (Fig. 1g-j, 2, Extended Data Fig. 4), we imaged the full stratum oriens dendritic arbour of n=12 CA1-PV+ interneurons from n=6 mice to investigate SPW-R-associated dendritic activities. We mapped n=58 dendrites with their surrounding neuropil. We analysed more than n=307 SPW-R events. Originally, we detected n=85 SPW-R-related dendritic dSpikes which has been extended to n=103, according to the Reviewer's request.<br>iii) In the third set of experiments (Fig. 3, 4, and Extended Data Fig. 12), we defined supralinear dSpikes associated with SPW-R doublets. We analysed the entire data pool of n=12 PV+ interneurons in n=6 mice, and we found SPW-R doublet-associated dSpikes in n=5 PV+ interneurons in n=5 mice.<br>iv) In the fourth set of experiments (Extended Data Fig. 5,6,8,15) we confirmed the in-vivo data through in-vitro measurements: (1) the GCamp6f sensor in n=10 mice, (2) specificity of uncaging-evoked doublet dSpikes in n=4 mice, (3) pharmacology in n=15 mice.
Randomization	Randomization was not performed because all mice were assigned to a single group.
Blinding	Blinding was not possible as experimental conditions were evident from the image data.

Reporting for specific materials, systems and methods

We require information from authors about some types of materials, experimental systems and methods used in many studies. Here, indicate whether each material, system or method listed is relevant to your study. If you are not sure if a list item applies to your research, read the appropriate section before selecting a response.

Materials & experimental systems

n/a	Involved in the study
☐	☒ Antibodies
☒	☐ Eukaryotic cell lines
☒	☐ Palaeontology and archaeology
☐	☒ Animals and other organisms
☒	☐ Human research participants
☒	☐ Clinical data
☒	☐ Dual use research of concern

Methods

n/a	Involved in the study
☒	☐ ChIP-seq
☒	☐ Flow cytometry
☒	☐ MRI-based neuroimaging

Antibodies

Antibodies used	rabbit anti PV-27 primary antibody (Swant, Switzerland, Lot. No. 2014, Cat# PV 27, RRID:AB 2631173, 1:1000), fluorochrome-conjugated secondary antibody 456 (Lifetech, A21207, donkey anti-rabbit Alexa 594, RRID: AB 141637, 1:500)
Validation	https://www.swant.com/pdfs/PV27_Rabbit_anti_Parvalbumin.pdf<br>https://www.thermofisher.com/antibody/product/Donkey-anti-Rabbit-IgG-H-L-Highly-Cross-Adsorbed-Secondary-Antibody-Polyclonal/A-21207

12

Animals and other organisms

Policy information about studies involving animals; ARRIVE guidelines recommended for reporting animal research

Laboratory animals	All experiments were conducted in accordance with the Animal Care and Experimentation Committee of the Institute of Experimental Medicine (approval reference numbers PE/EA/1517-7/2018). PV-Cre adult mice (6-24 weeks old) of both sexes were used (n=20 males, n=15 females) and were housed in a temperature-controlled environment (24±1°C) on a 12 h reverse light cycle (dark period between 08:00 and 20:00) and humidities between 40 and 70%. The animals were kept in small groups (2-4 mice/homecage) in enriched environment with cardboard rolls, rotary discs and extra nesting material (sizzle pet). During the training and the experimental period their water consumption was restricted to 1 ml/day after recovering from surgery. Weight loss due to water deprivation was kept below 20%. The mice had ad-libitum access to food. PV-GFP adult mice (6-24 weeks old) of both sexes were used (n=11 males, n=5 females) for in vitro electrophysiological and pharmacological experiments.
Wild animals	No wild animals were used in this study.
Field-collected samples	No field-collected samples were used in this study.
Ethics oversight	All experiments were conducted in accordance with the Animal Care and Experimentation Committee of the Institute of Experimental Medicine (approval reference numbers PE/EA/1517-7/2018).

Note that full information on the approval of the study protocol must also be provided in the manuscript.

dr Rózsa J Balázs

R
